# Proteasome Inhibitors Silence Oncogenes in Multiple Myeloma through Localized Histone Deacetylase 3 Stabilization and Chromatin Condensation

**DOI:** 10.1158/2767-9764.CRC-22-0255

**Published:** 2022-12-27

**Authors:** Laure Maneix, Polina Iakova, Shannon E. Moree, Joanne I. Hsu, Ragini M. Mistry, Fabio Stossi, Premal Lulla, Zheng Sun, Ergun Sahin, Sarvari V. Yellapragada, André Catic

**Affiliations:** 1 Huffington Center on Aging, Baylor College of Medicine, Houston, Texas.; 2 Stem Cells and Regenerative Medicine Center, Baylor College of Medicine, Houston, Texas.; 3 Department of Molecular and Cellular Biology, Baylor College of Medicine, Houston, Texas.; 4 Cell and Gene Therapy Program at the Dan L. Duncan Comprehensive Cancer Center, Baylor College of Medicine, Houston, Texas.; 5 Integrated Microscopy Core and GCC Center for Advanced Microscopy and Image Informatics, Baylor College of Medicine, Houston, Texas.; 6 Department of Hematology-Oncology, Baylor College of Medicine, Houston, Texas.; 7 Michael E. DeBakey Veterans Affairs Medical Center, Houston, Texas.

## Abstract

Proteasome inhibitors have become the standard of care for multiple myeloma. Blocking protein degradation particularly perturbs the homeostasis of short-lived polypeptides such as transcription factors and epigenetic regulators. To determine how proteasome inhibitors directly impact gene regulation, we performed an integrative genomics study in multiple myeloma cells. We discovered that proteasome inhibitors reduce the turnover of DNA-associated proteins and repress genes necessary for proliferation through epigenetic silencing. Specifically, proteasome inhibition results in the localized accumulation of histone deacetylase 3 (HDAC3) at defined genomic sites, which reduces H3K27 acetylation and increases chromatin condensation. The loss of active chromatin at super-enhancers critical for multiple myeloma, including the super-enhancer controlling the proto-oncogene c-MYC, reduces metabolic activity and cancer cell growth. Epigenetic silencing is attenuated by HDAC3 depletion, suggesting a tumor-suppressive element of this deacetylase in the context of proteasome inhibition. In the absence of treatment, HDAC3 is continuously removed from DNA by the ubiquitin ligase Seven in Absentia Homolog 2 (SIAH2). Overexpression of SIAH2 increases H3K27 acetylation at c-MYC–controlled genes, increases metabolic output, and accelerates cancer cell proliferation. Our studies indicate a novel therapeutic function of proteasome inhibitors in multiple myeloma by reshaping the epigenetic landscape in an HDAC3-dependent manner. As a result, blocking the proteasome effectively antagonizes c-MYC and the genes controlled by this proto-oncogene.

**Significance:**

Integrative genomics reveals that a key function of proteasome inhibitors involves limiting the activity of MYC and MYC-dependent genes through epigenetic repression.

## Introduction

Multiple myeloma, a cancer of terminally differentiated plasma cells, is the second most prevalent hematological malignancy (1). In the United States, there were an estimated 34,920 new multiple myeloma cases and 12,410 projected deaths in 2021 (1). Although survival has improved over the past two decades due to new drugs, immunotherapies, and the implementation of autologous stem cell transplantations (2, 3), multiple myeloma remains an incurable disease.

The standard of care for patients with multiple myeloma includes the use of proteasome inhibitors such as bortezomib (Velcade), carfilzomib (Kyprolis), and ixazomib (Ninlaro; refs. 4–6). Proteasome inhibitors interfere with the ubiquitin–proteasome system (UPS), the major proteolytic pathway by which cells regulate specific protein degradation. These inhibitory agents block selective protein elimination and regulate intracellular protein turnover. In humans, the UPS enlists a multi-step process that involves two ubiquitin-activating enzymes (E1s), which activate ubiquitin and transfer it to one of the 39 E2 ubiquitin–conjugating enzymes (7). The specificity and substrate selectivity of the ubiquitin-conjugating system are conferred by 600–700 E3-ubiquitin ligases, which in most cases attach ubiquitin to available amino residues, usually lysine side chains, on their substrate. Polyubiquitin chains conjugated through K48 generally target a protein for destruction by the proteolytic core of the UPS, the proteasome (8).

Proteasome inhibitors act through multiple mechanisms to promote cell death, including inhibition of nuclear factor-κB (NF-κB) signaling, activation of the c-Jun N-terminal kinase (JNK) pathway, and induction of the unfolded protein response pathway via endoplasmic reticulum stress (9). Gene expression is another function that depends on proteasome activity. Transcription factors and epigenetic regulators are short-lived proteins (10, 11). Transcription is highly dynamic and involves the constant surveillance and removal of transcriptional and epigenetic regulators by the UPS (12, 13). In response to various types of stimuli, E3-ubiquitin ligases direct the proteolytic removal of DNA-bound regulators, allowing for rapid modulation of gene expression and subsequent cellular adaptations. In addition, the location of DNA-associated proteins is carefully controlled in the nucleus and proteolytic elimination of these proteins at defined genomic regions ensures spatial specificity of degradation (14–16).

Although gene regulation and protein degradation are connected, one of the least understood features of proteasome inhibitors is how they interfere with transcription in a clinically relevant manner. In this study, we investigated the genome-wide changes triggered by proteasome inhibition on gene regulation in multiple myeloma. To determine how proteasome inhibition directly impacts transcriptional dynamics, we defined genomic sites of protein turnover and examined immediate transcriptional and epigenetic changes in multiple myeloma cells. Our results indicate that proteasome inhibitors repress oncogenic genes, including *c-MYC*, by increasing promoter and super-enhancer condensation.

## Materials and Methods

### Cell Lines

Human multiple myeloma cell lines MM.1S, MOLP-8, and U266.B1 were cultured in Roswell Park Memorial Institute (RPMI) 1640 medium (Hyclone, Cytiva) supplemented with 10% FBS (GenDEPOT, F0900–050), 100 U/mL penicillin/streptomycin (Gibco, 15140–122), 4.5 g/L glucose (Sigma, G8769), 1 mmol/L sodium pyruvate (Gibco, 11360070) and incubated at 37°C in a humidified atmosphere with 5% CO_2_. MM.1S (ATCC #CRL-2974, RRID:CVCL_8792) and U266.B1 (ATCC #TIB-196, RRID:CVCL_0566) cell lines were purchased from the ATCC and MOLP-8 cells were obtained from the German Collection of Microorganisms and Cell Cultures repository (DSMZ, ACC 569, RRID:CVCL_2124) in September 2015. HEK293T/17 (ATCC #CRL-11268, RRID:CVCL_1926) and HeLa (ATCC #CCL-2, RRID:CVCL_0030) cell lines were obtained from the Baylor College of Medicine Molecular and Cellular Biology Tissue Culture Core Laboratory in June 2015, and were initially purchased from ATCC by the Core. HEK293T cells and HeLa cells were cultured in DMEM (Corning 10–017-CV) supplemented with 10% FBS and 100 U/mL penicillin/streptomycin and grown in a 37°C incubator with a humidified atmosphere of 5% CO_2_. Cell lines were passaged less than 30 times (<6 months) and monitored for signs of bacterial or *Mycoplasma* contamination (MycoAlert kit, Lonza, LT07–318). Manufacturers performed authentication through short tandem repeat profiling.

The MM.1S-shHDAC3–inducible cell line was generated by lentiviral transduction using the pINDUCER11 (miR-RUG) vector system (Addgene plasmid #44363, RRID:Addgene_44363) and HDAC3 knockdown was induced in nonsilencing and shHDAC3 cells by treatment with 2 μg/μL doxycycline hyclate for 48 hours. pINDUCER11 (miR-RUG) was a gift from Dr. Thomas Westbrook (Baylor College of Medicine, Houston, TX) (17). MM.1S-SIAH2 stable cell line was generated using the pRetroX-IRES-ZsGreen1 retroviral vector (Takara Biosciences, 632520) encoding the human Seven in Absentia Homolog 2 (SIAH2) protein. Viruses were prepared in HEK293T/17 cells. After virus concentration with Lenti-X Concentrator (Takara, 631231), MM.1S cells were transduced with lentiviral or retroviral vector particles diluted in RPMI1640 media and 2 μg/mL of polybrene infection reagent (Millipore Sigma, TR-1003-G). Four days posttransduction, stable cell lines were analyzed by FACS on a BD FACSAria I sorter with gating based on forward/side scatter and events in the top 25% of GFP fluorescence were sorted at 100% purity. For lentiviral/retroviral transductions or transient transfections, a plasmid DNA containing the empty pRetroX-IRES-ZsGreen1 vector or a nontargeting pINDUCER11 construct served as controls.

### Proteasome Inhibitors

The FDA-approved proteasome inhibitors bortezomib and carfilzomib were purchased from Selleckchem (PS-341 and PR-171, respectively). While bortezomib is a reversible inhibitor of the 20S proteasome's β1- and β5-subunits, carfilzomib irreversibly binds to the β5-subunit and inhibits its chymotryptic-like activity (18). Lactacystin, an irreversible inhibitor of the β2- and β5-subunits of the 20S proteasome core, was obtained from Cayman Chemical (70980). Because of its capacity to bind to all three catalytic proteasome subunits, lactacystin is a more potent inhibitor than clinically approved proteasome inhibitors (19). Bortezomib and lactacystin share the same transcriptional target genes in MM.1S cells, as demonstrated by the almost perfect correlation (*R*^2^ = 0.9983) in distribution of FPKM counts in RNA sequencing (RNA-seq) following treatment with the two inhibitors. Both drugs showed similar changes based on individual genes and gene ontologies. No unique gene ontologies were altered specifically by either drug.

### Chromatin Immunoprecipitation Assays

Chromatin immunoprecipitation (ChIP) assays were carried out according to an optimized version of the protocol provided with the iDeal ChIP-seq Kit for Transcription Factors (Diagenode). Following proteasome inhibition for 3 hours with 25 μmol/L lactacystin (16), 60 nmol/L bortezomib, 60 nmol/L carfilzomib, or 0.1% v/v DMSO control, MM.1S, MOLP-8, MM.1S-SIAH2, MM.1S-shHDAC3, and control cells were collected in 50 mL conical tubes, counted, and assessed for cell viability. For each experimental condition, 25 million cells were washed once in sterile 1× PBS, resuspended in 1% formaldehyde in PBS solution, and fixed for 10 minutes at room temperature with gentle end-over-end rotation on a Hula mixer (10 rpm, Thermo Fisher Scientific).

Cross-linking reaction was quenched by adding glycine at a final concentration of 125 mmol/L to the fixation solution for additional mixing (5 minutes, 10 rpm). After incubation, fixed samples were washed once in sterile 1× ice-cold PBS and stored on ice until further processing. Cell lysis was carried out with ice-cold lysis buffers iL1b and iL2, according to manufacturer's protocol for suspension cells. After centrifugation, nuclear pellets were resuspended in SDS-containing shearing buffer iS1b supplemented with protease inhibitor cocktail at a concentration of 1.5 million cells per 100 μL buffer iS1b. Nuclear cell suspension was split into 250 μL aliquots and chromatin was sheared using a Bioruptor Pico water bath sonicator (Diagenode). To ensure generation of 150–300 bp DNA fragments suitable for next-generation sequencing (NGS), the Bioruptor Pico sonicator was set at 10 cycles, each cycle 30 seconds “ON” and 30 seconds “OFF”, and kept at 4°C. After sonication, samples were centrifuged (10 minutes, 16,000 × *g*, 4°C) to remove nuclear membrane debris and insoluble fraction. Supernatant (sonicated chromatin) was stored at −80°C into “Input” aliquots or used immediately in immunoprecipitation (IP) reactions, which were carried out with 2 μg of antibodies/IP and DiaMag Protein A–coated magnetic beads (30 μL/IP) under constant rotation on a Hula mixer (10 rpm). The ChIP-grade primary antibodies used in this study were the following: anti-H3K4me1 antibody (Abcam, ab8895, RRID:AB_306847), anti-H3K4me3 antibody (Abcam, ab8580, RRID:AB_306649), anti-H3K27ac antibody (Diagenode, C15410196, RRID:AB_2637079), anti-HDAC1 antibody (Active Motif, 40967, RRID:AB_2614948), anti-HDAC2 antibody (Active Motif, 39533, RRID:AB_2614959), anti-HDAC3 antibody (Millipore Sigma, 17–10238, RRID:AB_11213922), normal rabbit IgG isotype control (Cell Signaling Technology, 2729S, RRID:AB_1031062), and normal mouse IgG isotype control (Millipore Sigma, 17–10238, RRID:AB_11213922).

After immunoprecipitation overnight at 4°C, the immunoprecipitated complexes were captured with a magnetic rack, washed, and eluted according to Diagenode's protocol. ChIP and “Input” samples were then de-crosslinked overnight at 65°C in a temperature-controlled water bath. The next day, ChIP and “Input” DNA was recovered and purified using iPure beads provided in the manufacturer's kit. Finally, a 55%-25% DNA fragment double size selection was performed using Agencourt AMPure XP beads (Beckman Coulter) and final amounts of size-selected DNA were measured on a Qubit fluorometer (Thermo Fisher Scientific). The purified and size-selected DNA was then subjected to real-time qPCR or included in library preparation for NGS.

### Cleavage Under Targets and Release Using Nuclease

Cleavage Under Targets and Release Using Nuclease (CUT & RUN) was performed with a CUT&RUN assay kit according to the manufacturer's specifications (Cell Signaling Technology, 86652). Protocol optimization was based on the procedure described in a previous ChIP-seq study published by the authors’ group which examined degradative poly-ubiquitination sites in mouse embryonic fibroblast cells (16). Briefly, 250,000 MM.1S cells were used per condition and lysed in 4% digitonin. Capturing of DNA/protein complexes was performed with the following antibodies: 2 μL of anti-MYC antibody (Cell Signaling Technology, 13987, RRID:AB_2631168) per condition, 2 μL of anti-FLAG antibody (Sigma-Aldrich, Clone M2, F1804, RRID:AB_262044) per condition for MM.1S cells stably transduced with 3xFLAG-ubiquitin, and as a negative control 5 μL of rabbit IgG isotype control (Cell Signaling Technology, 66362) per condition.

### NGS (ChIP-seq, RNA-seq, and CUT & RUN)

#### ChIP-seq

Single-indexed DNA libraries were constructed with the Ultra Next DNA library prep kit I and II (New England Biolabs (NEB), E7370S and E7645S) and prepared for multiplex sequencing using NEBNext Multiplex oligos (E7335S and E7500S) following manufacturer's instructions. Library quality control, including assessment of fragment size distribution and quantification by qPCR, and library sequencing was conducted by the Baylor College of Medicine Genomic and RNA Profiling Core as previously published (20). Briefly, 1.8 pmol/L of equimolarly pooled libraries with 1% PhiX control spike-in were loaded onto a NextSeq 500 high output v2.5 flow cell (Illumina, 20024906) and analyzed on a Illumina NextSeq 500 sequencing system. The flow cell was sequenced in a 75-bp single-end run, enabling the generation of a minimum of 25 million reads per sample. DNA libraries prepared for sequencing on a Illumina HiSeq 2500 were processed identically for quality control. After equimolar pooling of the individual samples, a 10 pmol/L library with 5% PhiX control spike-in was sequenced on a Illumina HiSeq 2500 instrument as a 1 × 50 bp single-end sequencing run (25 million reads per sample) in rapid run mode (v.2). Clustering and sequencing performance were controlled as previously described (20).

To build the ChIP-seq heatmap showing the sensitivity of the three studied histone modifications to proteasome inhibitor lactacystin, histone mark binding levels for each chromosomal location were averaged from two independent ChIP-seq runs, median-corrected, and represented as log_2_ fold change (lactacystin-treated vs. control) of enriched ChIP-seq peaks.

#### RNA-seq

Transcript levels were evaluated six hours after treatment with 6 μmol/L lactacystin or 60 nmol/L bortezomib and compared with mock-treated MM.1S cells (DMSO at 0.1% v/v) (16). First, total RNA was isolated with the RNeasy Plus Mini kit (Qiagen, 74134) with additional on-column DNase I digestion (Qiagen, 79254). Then, sequencing libraries preparation was performed with the KAPA stranded RNA-seq kit with RiboErase (HMR; Roche, KK8483), including ERCC ExFold RNA spike-in mixes (Thermo Fisher Scientific, 4456739) to assess the platform dynamic range (Lactacystin #3 and bortezomib datasets only). Custom-designed indexed adapters were synthesized by Integrated DNA Technologies. The Genomic and RNA Profiling Core performed RNA-seq library quality controls and quantified multiplexed libraries by qPCR as described (20). Equimolarly pooled RNA-seq library products were diluted to 20 pmol/L for cluster generation by bridge amplification and sequenced onto a HiSeq 2500 sequencing instrument (Illumina) in rapid run mode (v2). PhiX Control v3 adapter-ligated library (Illumina, FC-1103001) was spiked-in at 2% by weight to ensure balanced diversity and to monitor clustering and sequencing performance. The paired-end run (2 × 100 bp) produced a minimum of 50 million reads per sample. Gene expression was normalized and quantified as FPKM (Fragments Per Kilobase per Million) using Cufflinks (RRID:SCR_014597) and Cuffdiff v.2.1.1 (RRID:SCR_001647) (21).

#### CUT & RUN

Single-indexed DNA libraries were prepared with the Ultra Next DNA library prep kit II (NEB, E7645S) and multiplexed for sequencing using NEBNext Multiplex oligos (E7500S). The protocol for DNA library preparation was adapted from previous publications and specifically optimized for CUT & RUN samples (22, 23). Library quality control was conducted by the Baylor College of Medicine Genomic and RNA Profiling Core as described in the “ChIP-seq” paragraph above. Then, pooled libraries were loaded onto a NextSeq 500 high output v2.5 flow cell (Illumina, 20024906) and analyzed on a Illumina NextSeq 500 sequencing system. The flow cell was sequenced in a 75-bp paired-end run, enabling the generation of a minimum of 19 million reads per sample. Clustering and sequencing performance were controlled as previously described (20).

All ChIP-seq and RNA-seq bioinformatic analyses were performed in-house with Linux command line tools. The workflow for fastq sequence data generation, sample demultiplexing, quality analysis of sequencing data and data processing with bioinformatics tools and algorithms was described (20). Gene ontology enrichment analysis was performed using DAVID version 6.8 annotation tool (RRID:SCR_001881, https://david.ncifcrf.gov/; ref. 24). ChIP-seq and CUT & RUN tracks were visualized with the Integrative Genome Viewer (IGV, Broad Institute, RRID:SCR_011793; ref. 25). The RNA-seq heatmap of select genes presented in the supplements was built with GraphPad Prism version 9.1.

### ChIP-qPCR Analysis

For qPCR analysis of the precipitated ChIP DNA, 0.5 μL of size-selected DNA and 2%–4% input material were used as template in PCR reactions performed with 10 μL of SYBR Green PCR Master Mix (Applied Biosystems, 4309155) and 1 μL of forward or reverse primers (20 μmol/L) in a total volume of 20 μL. Human negative control primer set 1 (Active Motif, 71001) was used as a negative control locus. Isotype negative controls (normal rabbit IgG or normal mouse IgG) were included in the experiment. The PCR amplification was carried out on a CFX96 real time PCR machine (Bio-Rad). The enrichment was determined with the percent input method, where amplification signals obtained from ChIP samples are divided by signal obtained from the input sample. The following qPCR primers were custom-designed with Primer-BLAST (National Center for Biotechnology Information) to amplify the promoters of interest:

- *AURKB* promoter: F: 5′-CGGACCCTCTGATCTACCT-3′, R: 5′-GAGAGTAGCAGTGCCTTGGA-3′;
- *AKAP1* promoter: F: 5′-GGTTGACCCTTCGAGACAAG-3′, R: 5′-GTCTACAGCGCTGGGCTAAC-3′;
- *CENP-C* promoter: F: 5′-ATTTCCTTCTCCCCAGCCTC-3′, R: 5′-GATTCGTTTCTTGCTCGGCT-3′;
- *MAD2L1* promoter: F: 5′-CTACTGAGCCGTCACGACTC-3′, 5′-GTGGCCGAGTTCTTCTGTAAG-3′.

### mRNA Quantitation by qRT-PCR

MM.1S, MOLP-8, U266.B1, and MM.1S-shHDAC3 cells were grown for 6 hours in presence or absence of 6 μmol/L lactacystin or 60 nmol/L bortezomib. Carfilzomib was added to MM.1S or MOLP-8 cells for 6 hours at a concentration of 20 nmol/L or 15 nmol/L, respectively. Untreated MM.1S-HDAC3 cells, MM.1S-SIAH2 cells, and corresponding control cells were harvested during exponential growth phase. Total RNA was extracted using the RNeasy Plus Mini kit (Qiagen, 74134) according to the manufacturer's protocol. RNA purity was verified by UV absorbance measurements at 260 and 280 nm on a NanoDrop 1000 (Thermo Fisher Scientific). qRT-PCR was performed on the isolated RNA with the SuperScript III Platinum SYBR Green One-Step Kit (Invitrogen, 11746–500) as recommended by the manufacturer, on a Bio-Rad CFX96 real time PCR instrument. Relative mRNA expression was calculated with the comparative *C*_t_ method (ΔΔ*C*_t_ method; ref. 26) and normalized using GAPDH expression levels as reference.

The following primers were used for qRT-PCR assays:

- *AURKB*: F: 5′-CAGTGGGACACCCGACATC-3′, R: 5′-GTACACGTTTCCAAACTTGCC-3′;
- *MAD2L1*: F: 5′-ATCACAGCTACGGTGACATTTC-3′, R: 5′-GCGGACTTCCTCAGAATTGGT-3′;
- *CENP-C*: F: 5′-TGGCAACTGATGTTAGTTCCAAA-3′, R: 5′-GGTGAGCCAACGGATAAGTAAA-3′;
- *AKAP1*: F: 5′-TGTCTCGGGAGCATGTCTTG-3′, R: 5′-GCCGACTCGATGAACCTACTT-3′;
- *TFAM*: F: 5′-CGCTCCCCCTTCAGTTTTGT-3′, R: 5′-CCAACGCTGGGCAATTCTTC-3′;
- *HSPA6*: F: 5′-CAAGGTGCGCGTATGCTAC-3′, R: 5′-GCTCATTGATGATCCGCAACAC-3′;
- *c-MYC*: F: 5′-GTCAAGAGGCGAACACACAAC-3′, R: 5′-TTGGACGGACAGGATGTATGC-3′;
- *SIAH2*: F: 5′-CATCAGGAACCTGGCTATGG-3′, R: 5′-GGACGGTATTCACATATGTC-3′;
- *HDAC1*: F: 5′-CTACTACGACGGGGATGTTGG-3′, R: 5′-GAGTCATGCGGATTCGGTGAG-3′;
- *HDAC2*: F: 5′-CCGCATGACTCATAATTTGCTG-3′, R: 5′-ATTGGCTTTGTGAGGGCGATA-3′;
- *HDAC3*: F: 5′-TCTGGCTTCTGCTATGTCAACG-3′, R: 5′-CCCGGTCAGTGAGGTAGAAAG-3′;
- *GAPDH*: F: 5′-GGAGCGAGATCCCTCCAAAAT-3′; R: 5′-GGCTGTTGTCATACTTCTCATGG-3′.

### Proximity Ligation Assays

Following a 24-hour treatment with 0.5 μmol/L lactacystin, approximately 2.4 × 10^5^ MM.1S-Flagged SIAH2 cells per well were cytospinned, attached onto a glass bottom CELLview cell culture slide (543979, Greiner Bio-One) precoated with Cell-Tak Cell and Tissue Adhesive (Corning), and fixed in 4% formaldehyde for 10 minutes at room temperature. After two consecutive washes with 1× ice-cold PBS, the fixed cells were permeabilized with 1× PBS with 0.5% Triton X-100 for 7 minutes, washed once with 1× PBS, and blocked in 5% donkey serum for 30 minutes at room temperature.

To visualize *in situ* SIAH2–HDAC3 interactions, proximity ligation assays were performed on lactacystin-treated MM.1S-Flagged SIAH2 cells with the Duolink *in Situ* Red Starter Kit Mouse/Rabbit (DUO92101, Millipore Sigma), adapting the manufacturer's protocol for MM.1S cells. First, the Duolink blocking solution was applied to the cells for 1 hour at 37°C in a humidified chamber. Then, slides were incubated with paired primary antibodies (mouse monoclonal anti-FLAG antibody (Sigma-Aldrich, Clone M2, F1804, RRID:AB_262044) and rabbit polyclonal anti-HDAC3 antibody (Abcam, ab7030, RRID:AB_305708)) diluted in Duolink antibody diluent overnight at 4°C in a histochemistry staining tray. After incubation, the slides were washed twice in Duolink buffer A before addition of the diluted anti-mouse PLUS and anti-rabbit MINUS PLA secondary probes for 1 hour at 37°C in a preheated humidity chamber. Following two washes with buffer A, circularization of DNA connector oligonucleotides with PLA probes was achieved by a DNA ligase previously diluted at 25 U/mL in Duolink Ligation buffer for 30 minutes at 37°C. Then, samples were washed twice in Duolink buffer A under gentle shaking and DNA template was amplified with a diluted DNA polymerase solution (125 U/mL) for 1 hour and 40 minutes at 37°C in the dark. Finally, hybridization of detection probes to the amplified template was performed and samples were rinsed twice in 1× wash buffer B for 10 minutes and once in 0.01× wash buffer B for 1 minute at room temperature. Slides were mounted with Duolink *in Situ* mounting medium containing DAPI.

For each antibody, a negative control condition was included where only one antibody or no antibody was incubated with the PLA probes. Fluorescence was visualized at 100× magnification with a Celldiscoverer7 microscope (Zeiss) controlled by the ZEN Pro imaging software (Zeiss) and images were processed for background subtraction and orthogonal projection. The exposure time (800 ms for PLA signal and 32 ms for DAPI) and gain were maintained at a constant level for all samples and the experimenter was blinded to the identity of the samples during the PLA staining.

### Seahorse Extracellular Flux Analysis of Mitochondrial Respiration

The day prior running the XFp Cell Mito Stress Test, MM.1S and MOLP-8 parental cells were incubated with sublethal concentrations of proteasome inhibitors (0.5 μmol/L lactacystin or 3 nmol/L bortezomib) for 24 hours in RPMI1640 medium (Hyclone, Cytiva) supplemented with 10% FBS (GenDEPOT) and 1% penicillin Streptomycin (Gibco/Thermo Fisher Scientific). MM.1S-SIAH2 cells, MM.1S-shHDAC3 cells, and respective control cells were replenished with fresh supplemented RPMI1640 medium. In addition, XFp sensor cartridges were hydrated with XFp Calibrant (Agilent Technologies) according to manufacturer's protocol. On the day of the assay, XFp assay medium was freshly prepared by supplementing XFp basal RPMI1640 medium (Agilent Technologies) with 10 mmol/L glucose (Sigma-Aldrich), 1 mmol/L sodium pyruvate (Gibco/Thermo Fisher Scientific), and 2 mmol/L glutamine (Gibco/Thermo Fisher Scientific). Cells were gently harvested, washed three times with XFp supplemented assay medium, and seeded at 30,000 cells per well in 50 μL of warmed XFp assay medium in a Seahorse 8-well XFp cell culture microplate (Agilent Technologies) coated with Cell-Tak Cell and Tissue Adhesive (Corning) beforehand. Microplates were centrifuged (1 minute, 200 × *g*, slow acceleration, zero braking) to allow the cells to adhere at the bottom of the wells. After addition of 120 μL of XFp assay medium, cells seeded in microplates were preequilibrated at 37°C in a non-CO_2_ incubator for 1 hour to eliminate CO_2_ from the media that would interfere with pH measurements.

The mitochondrial oxygen consumption rate of cells was directly measured on a Seahorse XFp Extracellular Flux Analyzer (Agilent Technologies). Mitochondrial function was analyzed through the sequential injections of modulators of the mitochondrial electron transport chain into the injection ports of the hydrated sensor cartridge. Oligomycin (1 μmol/L), an inhibitor of complex V ATP synthase was injected first in the assay following basal measurements. Then, maximal mitochondrial respiration was triggered by the addition of the uncoupling agent carbonyl cyanide-4 (trifluoromethoxy) phenylhydrazone (FCCP, 2 μmol/L). Finally, mitochondrial respiration was shut down and nonmitochondrial respiration was determined by the addition of a mixture of rotenone (0.5 μmol/L), a complex I inhibitor, and antimycin A (0.5 μmol/L), a complex III inhibitor. Oxygen consumption data was exported into the Seahorse Wave Desktop software (Agilent) and normalized by performing microscopic cell count prior the metabolic stress assay.

### Western Blots

MM.1S-SIAH2 cells were treated for 24 hours with 0.5 μmol/L lactacystin in order to prevent SIAH2 proteosomal degradation and facilitate its detection. Harvested MOLP-8, MM.1S-SIAH2, MM.1S-shHDAC3, and corresponding control cells were lysed by resuspending cell pellets in RIPA buffer (Sigma, R0278) supplemented with 1% XPert Protease Inhibitor Cocktail (GenDEPOT, P3100–005). Lysed cell suspensions were incubated for 1 hour on ice with continuous vortexing every 15 minutes prior to removal of the insoluble fraction by centrifugation at 14,000 × *g* for 30 minutes at 4°C. Protein concentration in the supernatant fraction were determined with a Bradford protein assay (Bio-Rad, 500–00006), using BSA as a standard.

Twenty micrograms of proteins were resolved on a precast Any Kd Bio-Rad SDS-PAGE polyacrylamide gel (Bio-Rad, 4569033) and transferred for 7 minutes onto a polyvinylidene difluoride (PVDF) membrane using a Trans-Blot Turbo transfer system (Bio-Rad). Western-blot analysis against HDAC3 was carried out using a rabbit polyclonal anti-HDAC3 antibody (Abcam, ab7030, RRID:AB_305708) diluted 1:5,000 in 1× Tris-buffered saline-Tween 20 (TBST) with 3% nonfat dry milk, and incubated for 1 hour at room temperature. A goat anti-rabbit IgG HRP-conjugated secondary antibody (Abcam, ab6721, RRID:AB_955447) was applied at 1:3,000 dilution in 1× TBST with 1% nonfat dry milk and was incubated for 1 hour at room temperature before visualizing the HRP-conjugated proteins with the ECL Clarity Western substrate (Bio-Rad, 1705061) using a Bio-Rad ChemiDoc imaging system.

The 3X-Flagged SIAH2 protein was detected with a mouse monoclonal anti-FLAG antibody (Sigma-Aldrich, Clone M2, F1804, RRID:AB_262044) diluted 1:1,000 in 1× TBST with 5% nonfat dry milk, and incubated overnight at 4°C. A goat anti-mouse IgG conjugated with HRP (Abcam, ab97023, RRID:AB_10679675) was used as secondary antibody (1:6,000 dilution in 1× TBST for 2 hours at room temperature). Protein signals were detected with the SuperSignal West Pico PLUS chemiluminescent substrate (Thermo Fisher Scientific, 34577) and captured on a Bio-Rad ChemiDoc imager. A HRP-linked GAPDH recombinant antibody (Abcam, ab204481) or an anti-β-tubulin antibody (Cell Signaling Technology, 86298, RRID:AB_2715541) was used as a loading control.

For c-MYC detection, 5 μg of protein lysates were resolved on a SDS-PAGE gel and transferred onto a PVDF membrane as described above. First, the membrane was incubated with 5% BSA in TBST for 1 hour at room temperature to block nonspecific binding. Then, a rabbit monoclonal anti-c-MYC/N-MYC antibody (Cell Signaling Technology, 13987, RRID:AB_2631168) was added at a 1:1,000 concentration diluted in 5% BSA with TBST for 1 hour at room temperature. Immunoreactive proteins were detected with a goat anti-rabbit HRP–conjugated secondary antibody (Abcam, ab6721, RRID:AB_955447) using the ECL Clarity Western substrate kit (Bio-Rad, 1705061). The density and size of the protein bands were quantified in ImageJ (RRID:SCR_003070).

### Cell Proliferation Assays

Cells were plated at 7.5 × 10^5^ cells/well (MM.1S cells) or 4.5.10^5^ cells/well (MOLP-8 cells) in 6-well plates, allowed to recover for 24 hours before treatment with proteasome inhibitors (0.5 μmol/L or 1 μmol/L lactacystin for MM.1S cells and MOLP-8 cells, respectively; 3 nmol/L or 8 nmol/L bortezomib for MM.1S cells and MOLP-8 cells, respectively). Cells were checked for growth and viability after 2, 4, 6, and 8 days. MM.1S-SIAH2 cells and corresponding control cells were seeded at 4.5 × 10^5^ cells/well in 6-well plates, remained untreated during the duration of the assay, and were checked for growth and viability every day for 7 days. At each timepoint, cells were stained with acridine orange/propidium iodide dual-fluorescent dye (Via Stain AO/PI, Nexcelom, CS2–0106) as recommended by the manufacturer and cell counts and cell viability were measured on a Cellometer Auto 2000 automated cell counter (Nexcelom).

### Quantification and Statistical Analyses

All statistical analyses were performed using GraphPad Prism version 8 or 9.1 software (RRID:SCR_002798) or Microsoft Excel. Statistical analysis for individual gene analyses was performed using a two-tailed Student *t* test while large datasets were compared with a two-sided Gehan–Breslow–Wilcoxon rank-sum test, a Mann–Whitney test, or a Mantel–Cox log-rank test. Kaplan–Meier survival curves were tested for significance using both log-rank test and Gehan–Breslow–Wilcoxon test. For violin plots, the dashed line marks the median and the dotted lines represent the lower and upper quartiles. All data are representative of three or more experiments, unless otherwise specified in the legends.

### Data Availability

The data generated in this study are available within the article and its supplementary data files. Raw data generated in this study are available upon request from the corresponding author. All NGS datasets generated from this study have been deposited in publicly available repositories. For transcriptomic analysis of MM.1S cells treated with proteasome inhibitors, raw and processed RNA-seq datasets have been deposited with Gene Expression Omnibus (GEO, RRID:SCR_005012; Lactacystin #1 and #2 datasets; GEO accession number GSE193303) or have been previously published (lactacystin #3 and bortezomib datasets; ref. 27). All ChIP-seq and CUT & RUN data presented in this study have been deposited in the NCBI GEO database with accession number GSE193303.

For survival and primary transcript analyses, gene expression data and outcomes of patients with relapsed multiple myeloma enrolled on the Assessment of Proteasome Inhibition for Extending Remissions (APEX) phase II and phase III multicenter clinical trial of bortezomib (versus dexamethasone) were obtained from previously published Affymetrix microarray results (4, 28) and downloaded from the Gene Expression Omnibus (GEO) database with accession number GSE9782. For survival analyses in the supplements, gene expression data and survival outcomes of patients treated with multiple myeloma drug combination regimens (Total Therapy TT2 and TT3 treatments) were obtained from the previously published MicroArray Quality Control (MAQC)-II study (29) and downloaded from the GEO database with accession number GSE24080. *HDAC3* and *SIAH2* gene expression data in the supplements were subclassified according to tissue origin by analyzing RNA-seq data for 1457 cell lines available in the Cancer Cell Line Encyclopedia (CCLE, Broad Institute, https://portals.broadinstitute.org/ccle). Figure 6 was created with BioRender.com (RRID:SCR_018361).

## Results

### Genome-wide Effects of Acute Proteasome Inhibition on Transcription and Chromatin in Multiple Myeloma Cells

**FIGURE 1:**
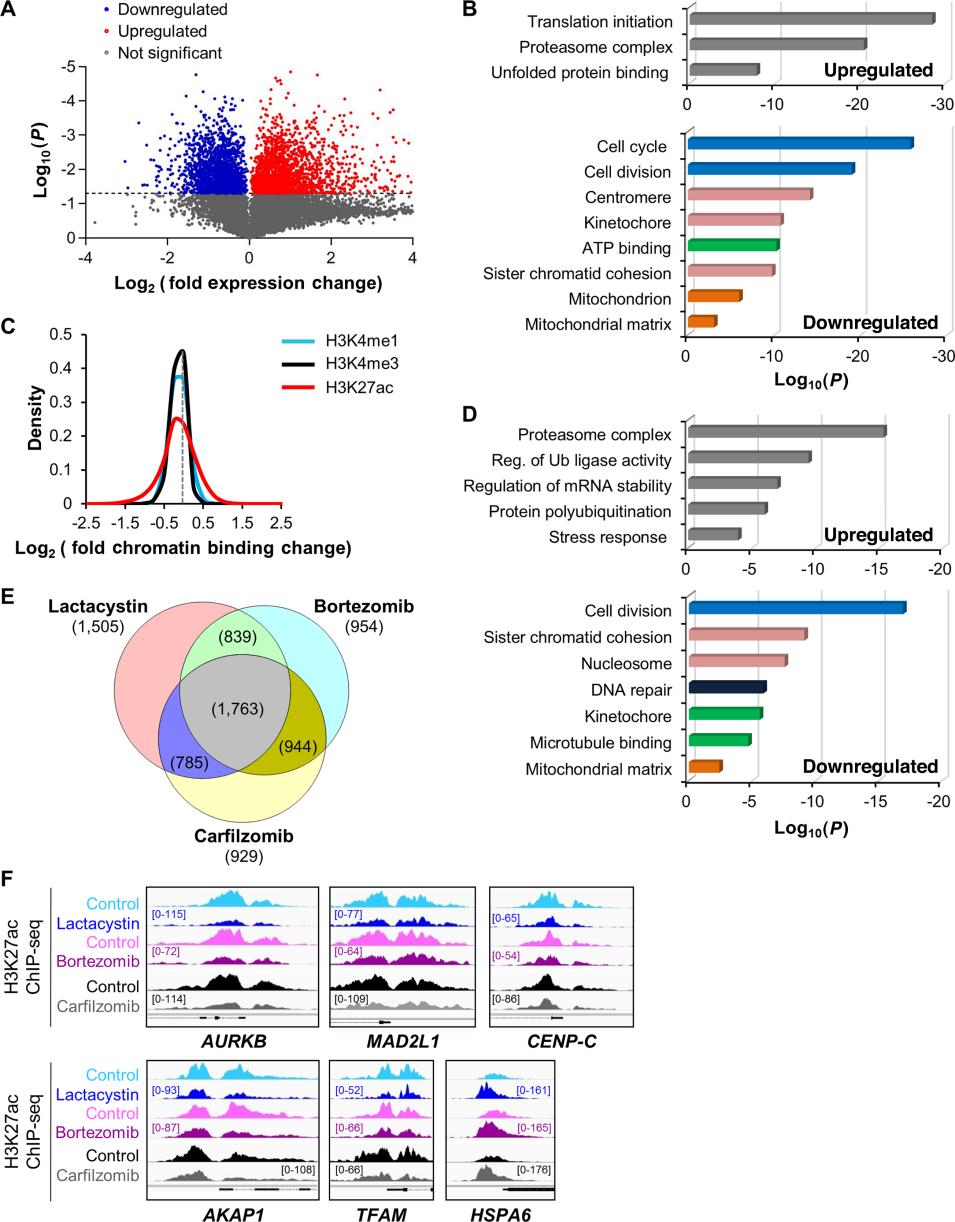
Proteasome inhibition represses H3K27 acetylation and transcription of genes involved in multiple myeloma growth and metabolism. **A,** Volcano plot representation of differential expression analysis of genes in control versus lactacystin-treated MM.1S cells measured by RNA-seq after 6 hours treatment. Blue and red dots mark the genes with significantly decreased or increased expression, respectively, in proteasome inhibitor–treated cells compared with control samples. The *P* values shown on the *y*-axis are based on paired Student two-tailed *t* test. **B,** Functional distribution of gene clusters upregulated (top) or downregulated (bottom) by proteasome inhibitor lactacystin as measured by RNA-seq. Differentially expressed RNAs were analyzed for significantly enriched functional annotation terms, as determined by DAVID. Transcription of cell growth and metabolic gene clusters was the most strongly repressed after treatment. **C,** Sensitivity of histone marks (H3K4me1, H3K4me3, and H3K27ac) to proteasome inhibitor lactacystin (3-hour treatment). Data show that the H3K27ac histone mark is more sensitive to proteasome inhibitor than H3K4me1 and H3K4me3 marks. Data are represented as log_2_ fold change (lactacystin-treated vs. control) of significantly enriched ChIP-seq peaks for the three studied histone modifications and are representative of two independent experiments. The gray dotted line intersects the *x*-axis at zero (no change). **D,** Functional distribution of gene clusters up- or downregulated after 3-hour treatment with proteasome inhibitor lactacystin. Gene activities (H3K27ac mark) that were upregulated (top) or downregulated (bottom) after lactacystin treatment were analyzed for significantly enriched functional annotation terms, as determined by DAVID. Data are representative of two independent experiments. Reg., regulation; Ub, ubiquitin. **E,** Venn diagram showing the overlap of repressed H3K27 acetylation sites within 1 kb of transcription start site in MM.1S cells treated with lactacystin, bortezomib, or carfilzomib. 1,763 genes showed repressed H3K27 acetylation with all three treatments. **F,** H3K27 acetylation was rapidly repressed in cell cycle (*AURKB*, *MAD2L1*, *CENP-C*), mitochondrial (*AKAP1*, *TFAM*), and stress response (*HSPA6*) gene promoters following treatment with lactacystin, bortezomib, and carfilzomib in MM.1S cells. In contrast, the stress response gene *HSPA6* displayed elevated H3K27 acetylation levels, indicating that the response to proteasome inhibition is gene-specific. The gene structure is shown in black at the bottom of each panel. All Integrative Genomics Viewer (IGV) ChIP-seq tracks in a given comparison are represented at the same scale (numbers in brackets at the *y*-axis). The genomic region on the *x*-axis spans 2.5 kb for all the regions. Images are representative of two independent experiments.

Transcription factors and epigenetic regulators are short-lived proteins (10), and proteasome inhibition is expected to significantly affect gene activity in multiple myeloma cells by slowing down the high turnover of these proteins. To verify this, we first analyzed the transcriptional response to acute proteasome inhibition in multiple myeloma cells. We treated the MM.1S cell line with the inhibitor lactacystin, which blocks all three active sites of the proteasome, and performed RNA-seq. The response pattern we observed was similar to treatment with the clinically approved drug bortezomib and we validated this response by qRT-PCR with the clinical drug carfilzomib (Fig. 1A; Supplementary Figs. S1A and S1B and S2; RNA-seq data also available as Supplementary Data). Upregulated gene ontologies included chaperones and other protein stress response factors. In contrast, treatment immediately repressed genes involved in mitochondrial activity and cell proliferation (Fig. 1B).

Given the variety of genes affected by proteasome inhibition, we next investigated whether treatment altered the epigenetic landscape of multiple myeloma cells. We determined the intensity of the anatomic chromatin marks H3K4me1 (enhancers), H3K4me3 (promoters), and the functional mark H3K27ac, which decondensates chromatin and increases accessibility of DNA (30–32). Following 3-hour lactacystin treatment of MM.1S cells, we performed ChIP and NGS to map epigenetic changes and identified unique gene clusters dynamically regulated by protein degradation. Our global ChIP-seq analysis of histone marks showed that enhancer and promoter marks (H3K4me1 and H3K4me3, respectively) only modestly responded to acute proteasome inhibition, while histone H3K27 acetylation was robustly up- or downregulated early after treatment with lactacystin (Fig. 1C; Supplementary Fig. S1C). About 14%–15% of enhancers and promoters were associated with higher acetylation, and 16%–18% showed lower acetylation after treatment (Supplementary Fig. S1D).

We performed gene ontology (GO) analyses of genes in which proteasome inhibition modulated gene expression and H3K27 acetylation to understand how treatment functionally impacts cellular pathways that might be relevant for the response of this cancer to these drugs. We found matching gene ontologies for the transcriptional and H3K27 acetylation response to proteasome inhibitors in MM.1S cells, indicating that elevated gene activity was driven by higher H3K27 acetylation, and reduced transcriptional output was caused by loss of H3K27 acetylation. As expected, proteasome inhibition upregulated H3K27 acetylation at stress response genes, including genes encoding the proteasome complex (Fig. 1D). Importantly, cell growth and metabolic gene clusters were strongly repressed at the transcript and H3K27 acetylation levels (Fig. 1B and D). We observed a strong overlap in genes with repressed H3K27 acetylation sites for each of the three proteasome inhibitors used in this study, indicating all three proteasome inhibitors repress similar target genes (Fig. 1E). The gene clusters that were the most down-regulated by proteasome inhibition were cell division, mitotic-related, and mitochondrial-related genes. For instance, blocking proteasome activity by either lactacystin or the clinically approved inhibitors bortezomib and carfilzomib led to a decrease in H3K27 acetylation at the promoters of cell cycle (Aurora Kinase B, *AURKB*; Mitotic Arrest Deficient 2 Like 1, *MAD2L1*; Centromere Protein C, *CENP-C*) or nuclear-encoded mitochondrial genes (A-Kinase Anchor Protein 1, *AKAP1*; Mitochondrial Transcription Factor A, *TFAM*; Fig. 1F). In contrast, H3K27 acetylation was increased at the promoters of stress response genes such as *HSPA6*. We validated these results by performing independent ChIP-qPCR assays in a separate MM cell line, MOLP-8 (Supplementary Fig. S1E). These results provide mechanistic insights into how proteasome inhibitors potentially act on proliferation and metabolism to slow disease progression in the clinic.

### Proteasome Inhibitors Repress Oncogene Transcription and Induce Chromatin Condensation at the *c-MYC* Super-enhancer

**FIGURE 2:**
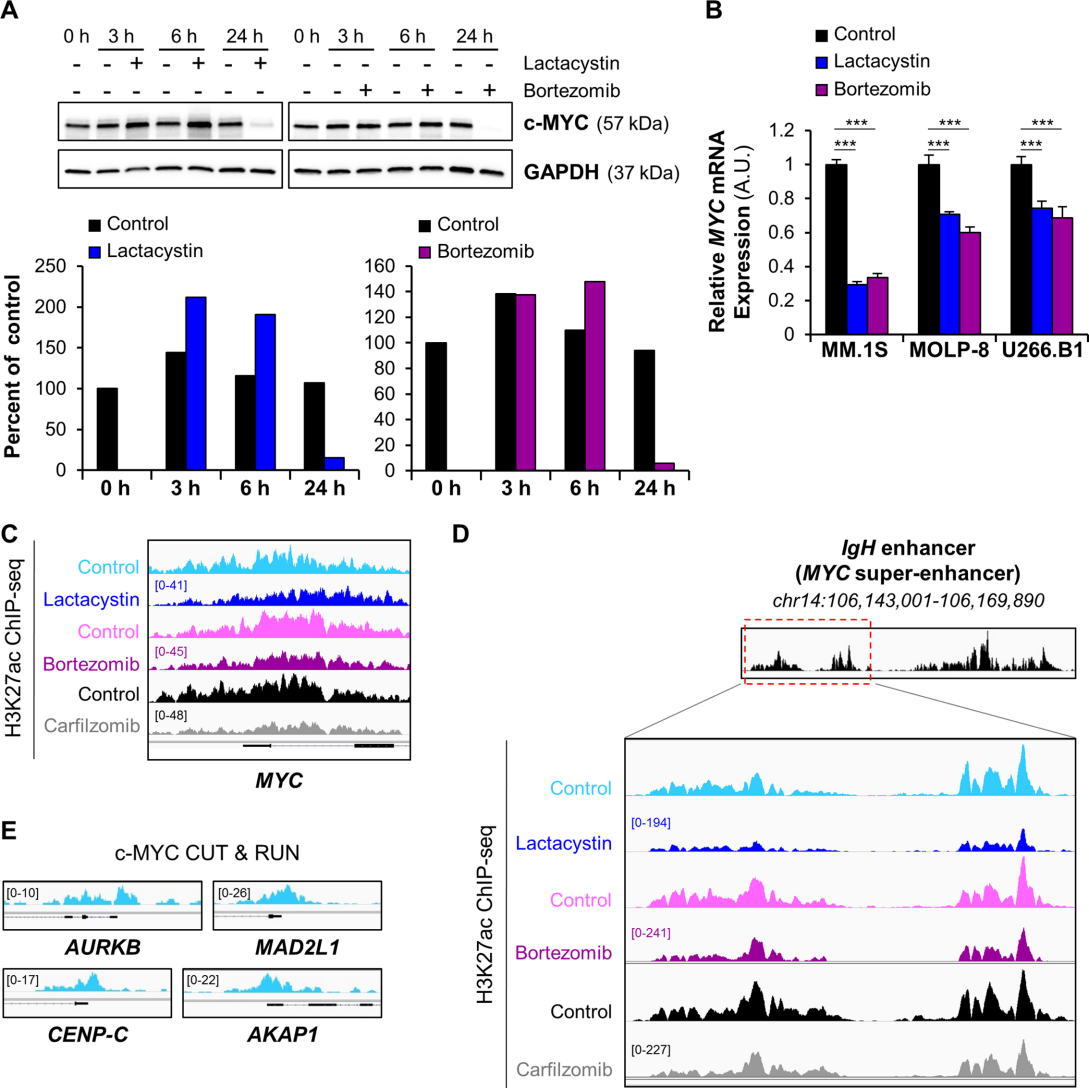
Proteasome inhibitors repress *c-MYC* gene expression and induce chromatin condensation at the *c-MYC* super-enhancer. **A,** Western blot analysis of c-MYC protein levels in MOLP-8 cells shows the biphasic effect of proteasome inhibitor treatment (12.5 μmol/L Lactacystin; 30 nmol/L Bortezomib) on c-MYC protein levels over time (3, 6, and 24 hours). GAPDH was used as an internal control. The relative expression of c-MYC protein was quantified in ImageJ and normalized to that of GAPDH protein. The levels of c-MYC protein at each timepoint were densitometrically compared and expressed as percent of the untreated 0-hour time point. Degradation of c-MYC at later timepoints can be attributed to incomplete inhibition of the proteasome and a bortezomib half-life of about 12 hours. **B,** qRT-PCR measurement of *MYC* mRNA levels in MM.1S, MOLP-8 and U266.B1 cells demonstrates that lactacystin or bortezomib transcriptionally repress the *c-MYC* oncogene after 6-hour treatment. ***, *P* < 0.001 determined by unpaired Student two-tailed *t* test. **C,** Representative ChIP-seq tracks of H3K27ac sites on *MYC* gene following exposure of MM.1S cells to different proteasome inhibitors show that H3K27 acetylation at the *c-MYC* promoter was rapidly repressed after treatment. The gene structure is shown in black at the bottom of the panel. IGV tracks in a given comparison are represented at the same scale (numbers in brackets at the *y*-axis). The genomic region on the *x*-axis spans 5 kb of the *MYC* gene. IGV snapshots are representative of two independent experiments. **D,** Gene tracks of H3K27ac ChIP-seq occupancy at *c-MYC* super-enhancer in MM.1S cells show that exposure to lactacystin, bortezomib, and carfilzomib reduces H3K27 acetylation of the super-enhancer, especially at its 5′-end (red dashed box). IGV tracks in a given comparison are represented at the same scale (numbers in brackets at the *y*-axis). The genomic region on the *x*-axis spans 9 kb of the *c-MYC* super-enhancer. IGV snapshots are representative of two independent experiments. **E,** CUT & RUN gene tracks of c-MYC–binding sites in MM.1S cells. The genomic region on the *x*-axis spans 2.5 kb for all the genes. IGV snapshots were the result of a single CUT & RUN experiment.

The proto-oncogene c-MYC controls growth-related genes, including cell-cycle factors and nuclear-encoded mitochondrial genes. Specifically, the activation of *c-MYC* is one of the key molecular events mediating disease progression from the early stage of monoclonal gammopathy of undetermined significance to multiple myeloma (33–35). As c-MYC is an exceptionally short-lived protein, proteasome inhibitors would be expected to stabilize it. Indeed, in the short term, we observed increased c-MYC protein in multiple myeloma cells in the presence of proteasome inhibitors. However, after several hours, c-MYC protein levels dropped (Fig. 2A). This surprising effect is driven by potent transcriptional repression of the *c-MYC* gene (Fig. 2B) and is likely precipitated by a rapid decrease in H3K27 acetylation at the *c-MYC* promoter following treatment (Fig. 2C). At the IgH locus, which corresponds to the *c-MYC* super-enhancer in MM.1S cells (36, 37), ChIP-seq assays showed that proteasome inhibition by lactacystin, bortezomib, or carfilzomib decreased H3K27 acetylation (Fig. 2D). These results suggest that proteasome inhibitors antagonize c-MYC activity on two levels: they directly repress expression of the *c-MYC* gene through H3K27 deacetylation at its promoter and super-enhancer, and reduce expression of c-MYC target genes (Fig. 2E) through H3K27 deacetylation at their promoters (Fig. 1F).

Alterations to histone modifications, followed by chromatin remodeling, can initiate changes in gene expression (38). Loss of H3K27 acetylation reduces the DNA accessibility and impedes transcription factor binding. To validate the downstream effects of genome-wide changes in acetylation on transcription following proteasome inhibition, we confirmed RNA-seq data by qRT-PCR in three independent multiple myeloma cell lines (MM.1S, MOLP-8, and U266.B1 cells). The inhibition of the proteasome activity by lactacystin, bortezomib, or carfilzomib significantly decreased mRNA expression levels of cell-cycle and mitochondrial genes in all three cell lines (Supplementary Fig. S2A–S2D).

To assess whether modulating the transcription of cell-cycle or mitochondrial genes could affect patient survival, we examined a gene expression dataset previously published as part of the APEX trial (4, 28) where the global transcriptome was analyzed in CD138^+^ multiple myeloma cells of 264 patients with relapsed and refractory disease. A survival analysis shows that low expression of mitochondrial or cell-cycle genes correlates with better survival (Supplementary Fig. S3A and S3B), suggesting that repression of mitochondrial or cell-cycle genes by proteasome inhibitors might be clinically relevant.

We next examined whether cell proliferation and oxidative metabolism were functionally affected in multiple myeloma cells treated with proteasome inhibitors. Sublethal doses of lactacystin or bortezomib for 8 days, significantly slowed cell growth (Supplementary Fig. S3C). In addition, multiple myeloma cells treated for 24 hours with sublethal concentrations of lactacystin or bortezomib showed significantly reduced rates of oxidative phosphorylation at baseline and maximum capacity (Supplementary Fig. S3D and S3E). Multiple myeloma cells rely on mitochondrial activity for ATP production to fuel immunoglobulin hyperproduction (39, 40). Our results suggest that inhibition of the proteasome functionally affects cell proliferation and energy metabolism through repression of relevant genes.

### Synergy Between Elevated HDAC3 and Proteasome Inhibition in Primary Multiple Myeloma

**FIGURE 3:**
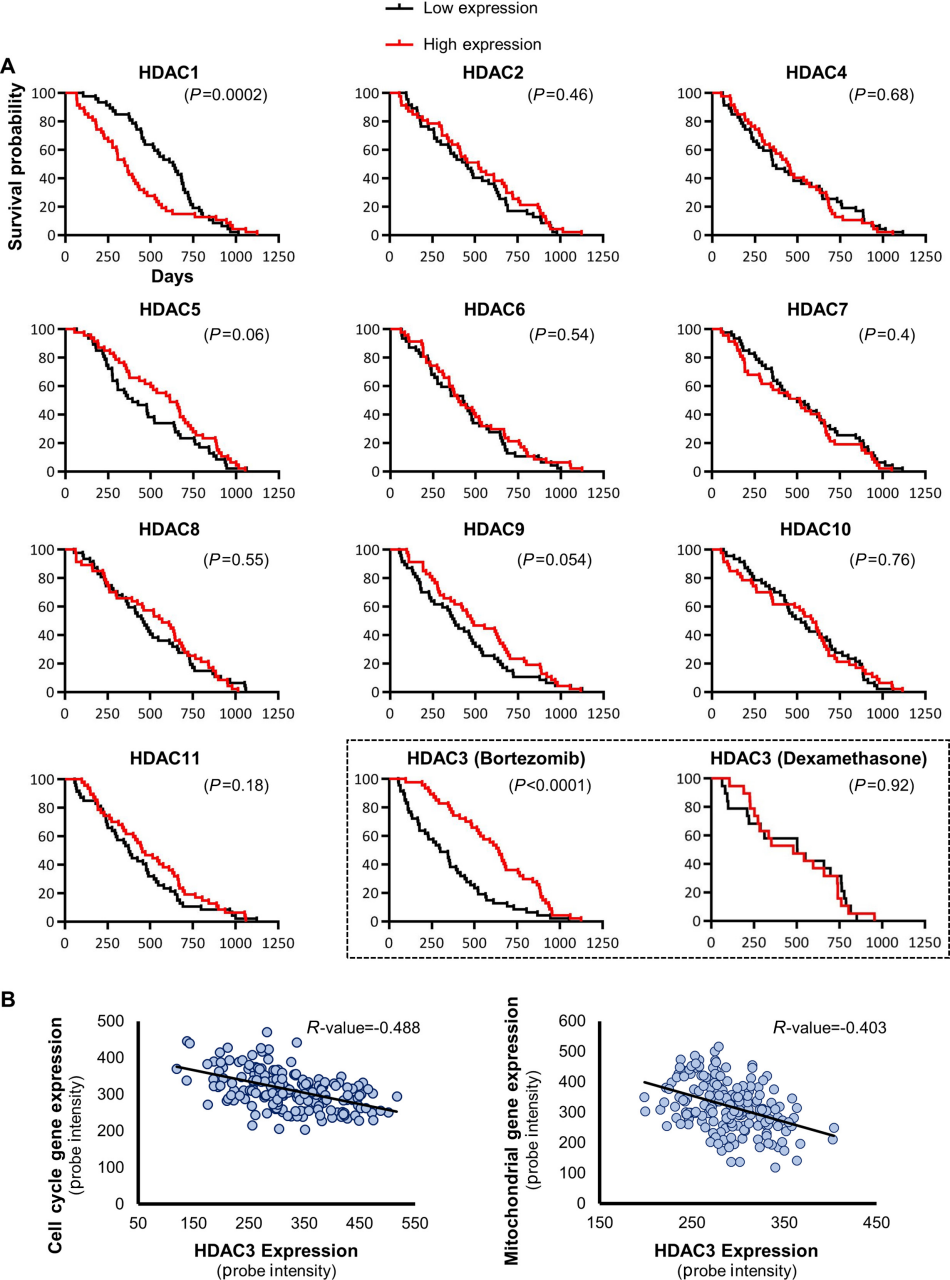
Tumor-suppressive effects of HDAC3 in primary multiple myeloma in combination with proteasome inhibitors. **A,** Kaplan-Meier representation of overall survival times of relapsed multiple myeloma participants enrolled in the APEX phase II and phase III clinical trial (4, 28) were compared on the basis of individual expression levels of 11 class I, II, and IV HDACs. Survival of patients expressing each HDAC ranked in the top versus bottom quarter was compared in the cohort receiving bortezomib treatment only. The two histone deacetylases that correlated significantly with overall survival in multiple myeloma were HDAC1, with a potentially oncogenic effect, and HDAC3 with a potentially suppressive effect. Notably, HDAC3 only correlated with better outcomes when expressed in the top quartile of the patient population that was subsequently treated with proteasome inhibitor bortezomib, not in patients in the dexamethasone control arm (black dashed box). In the dexamethasone cohort, the median survival time was 504 days and 481 days for the low expression and the high expression group, respectively. In the bortezomib cohort, the median survival time was 300 days and 641 days for the low expression and the high expression group, respectively. Results of the cohort receiving dexamethasone are not represented for the other HDACs. The transcript levels of HDAC genes were determined based on a DNA microarray study in primary CD138^+^ multiple myeloma cells (4, 28). The indicated *P* values were calculated with the Gehan–Breslow–Wilcoxon test and significance was verified with the log-rank test. **B,** Scatter diagram showing the anticorrelation between HDAC3 and cell cycle (left) or mitochondrial (right) gene expression in patients with multiple myeloma. A previously published DNA microarray study (4, 28) was analyzed to determine expression levels of the selected genes in primary CD138^+^ multiple myeloma cells. The human mitochondrial gene list was downloaded from the MitoCarta 3.0 database (43). The Pearson correlation coefficient (*R* value) is indicated for each scatter plot. The values of the *x*- and *y*-axis are RMA normalized Affymetrix probe intensity.

We next sought to investigate how proteasome inhibition causes H3K27 deacetylation in cell cycle and nuclear-encoded mitochondrial genes, and in the *c-MYC* super-enhancer. Histone deacetylases (HDAC) are a class of enzymes that can render the chromatin less accessible to transcription factors and coregulators and effectively silence gene transcription, by removing the acetyl groups from lysine residues of histone tails. The mammalian genome encodes 11 canonical HDAC isoforms (41, 42). To determine whether canonical histone deacetylase expression levels impact overall survival of patients with multiple myeloma, we analyzed the gene expression profiling dataset of 264 patients with relapsed or refractory multiple myeloma from the APEX study and assessed overall survival in dependence of HDAC expression (4, 28). While no significant survival difference was found for 9 of 11 canonical HDACs, HDAC3 was the only deacetylase associated with significantly better survival if expressed at high levels in patients treated with bortezomib (Fig. 3A). The survival advantage of HDAC3 was limited to patients who received treatment with bortezomib and was not apparent in the control set of patients who received dexamethasone. Considering that repression of cell-cycle and mitochondrial genes correlated with better survival, HDAC3 might be a candidate suppressor in a regulatory model where high expression of a histone deacetylase improves survival. We validated the impact of HDAC3 expression on overall survival, using a second independent dataset (Supplementary Fig. S3A). Furthermore, HDAC3 transcript abundance showed significant anticorrelation with the expression of cell-cycle and mitochondrial genes in these patients (Fig. 3B). These clinical data indicate that elevated HDAC3 levels might be beneficial to patient survival when combined with proteasome inhibition.

### Proteasome Inhibitors Stabilize HDAC3 in a DNA Site-specific Manner in Multiple Myeloma Cells

**FIGURE 4:**
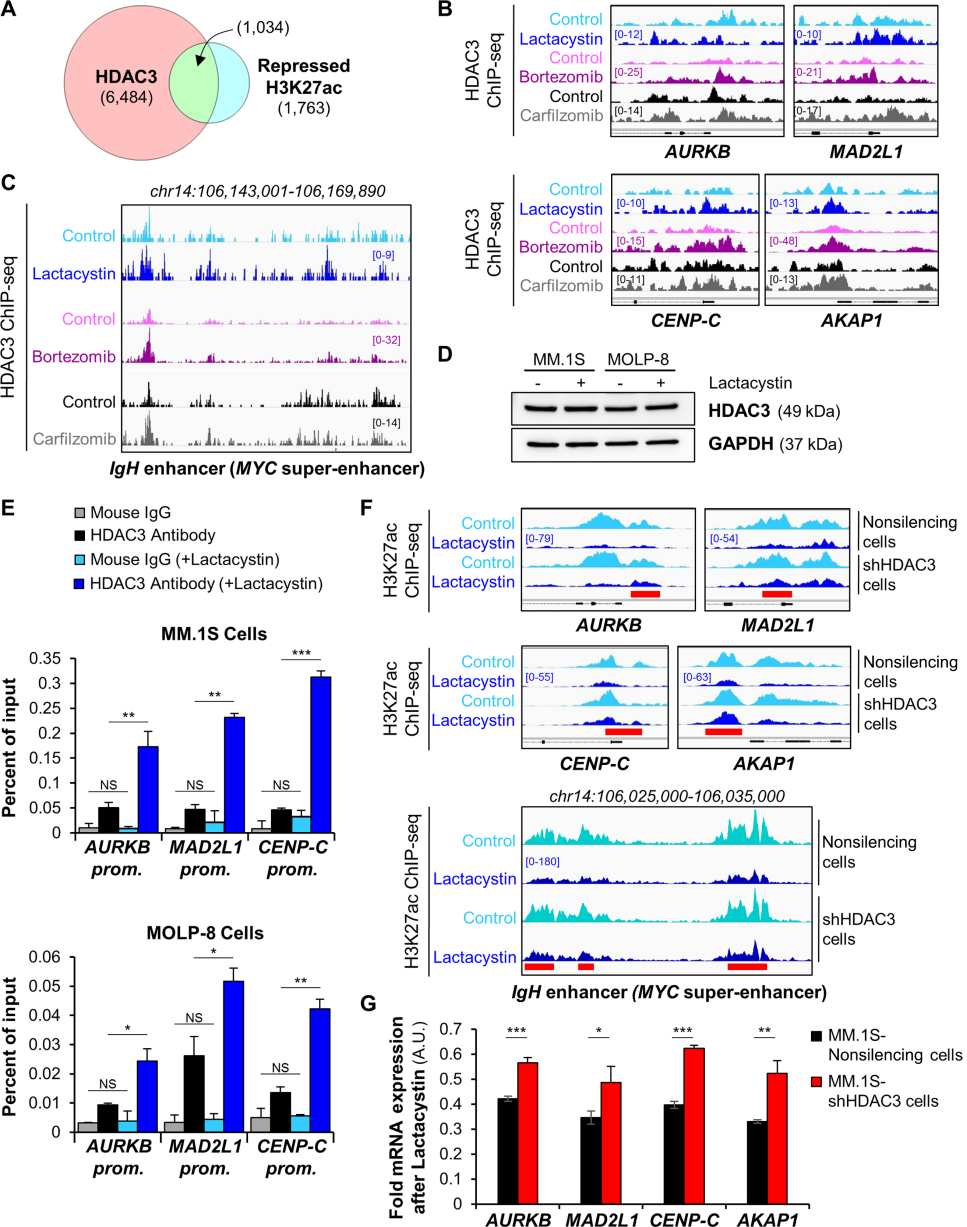
DNA site–specific stabilization of HDAC3 by proteasome inhibition. **A,** Venn diagram shows the overlap of HDAC3-associated sites and repressed H3K27 acetylation sites within 1 kb of transcription start site after treatment of MM.1S cells with proteasome inhibitors. Repressed acetylation sites were defined as overlapping H3K27ac sites which were reduced by all three treatments shown in Fig. 1E. **B,** Representative IGV browser ChIP-seq tracks of HDAC3-associated binding sites in MM.1S cells show elevated HDAC3 DNA occupancy at the promoters of cell-cycle and mitochondrial genes following proteasome inhibitor exposure. All IGV tracks in a given comparison are represented at the same scale (numbers in brackets at the *y*-axis). The gene structure is shown in black at the bottom of each panel. The genomic region on the *x*-axis spans 2.5 kb for all the genes. IGV snapshots are representative of two independent experiments. **C,** Gene tracks of HDAC3 ChIP-seq occupancy at the *c-MYC* super-enhancer in MM.1S cells following exposure to proteasome inhibitors. The HDAC3 sites that show marked increase of DNA occupancy levels within the super-enhancer matched with the H3K27 acetylation sites that are the most repressed following treatment (see Fig. 2D). IGV tracks in a given comparison are represented at the same scale (numbers in brackets at the *y*-axis). The genomic region on the *x*-axis spans 25 kb of the *c-MYC* super-enhancer. IGV snapshots are representative of two independent experiments. **D,** Western blot analysis of HDAC3 expression levels in MM.1S cells and MOLP-8 cells shows that proteasome inhibition does not affect global cellular levels of HDAC3 protein after treatment with 6 μmol/L or 0.5 μmol/L lactacystin for 6 or 24 hours, respectively. GAPDH was used as an internal control. **E,** HDAC3 was locally stabilized following proteasome inhibition. ChIP-qPCR analysis of HDAC3 DNA occupancy at selected promoters following proteasome inhibition with lactacystin in MM.1S cells (top) and MOLP-8 cells (bottom). NS, not significant; ***, *P* < 0.001; **, *P* < 0.01; and *, *P* < 0.05 determined by unpaired Student two-tailed *t* test. **F,** H3K27 acetylation is sensitive to proteasome inhibition and HDAC3 expression. Representative IGV browser ChIP-seq tracks of H3K27ac peaks following lactacystin exposure of MM.1S-shHDAC3 knockdown cell line compared with scrambled control demonstrates that loss of H3K27 acetylation following proteasome inhibition is mediated by HDAC3 and attenuated in HDAC3 knockdown cells. The red bars indicate regions in which the flattening of the H3K27 acetylation landscape by proteasome inhibitors is attenuated by HDAC3 knockdown. Peaks are more accentuated in knockdown cells and summits are up to 40% higher. For each gene panel, all IGV tracks are represented at the same scale (numbers in brackets at the *y*-axis). The gene structure is shown in black at the bottom of each panel. The genomic region on the *x*-axis spans 2.5 kb for the four genes in the top panel. IGV snapshots are representative of two independent experiments. **G,** Transcriptional repression by proteasome inhibitors is attenuated in HDAC3 knockdown cells. qRT-PCR analysis of cell-cycle and mitochondrial genes after 6-hour treatment of MM.1S-shHDAC3 or scrambled control cells with 6 μmol/L lactacystin. ***, *P* < 0.001; **, *P* < 0.01; and *, *P* < 0.05 determined by unpaired Student two-tailed *t* test.

Our observations imply that HDAC3 can slow multiple myeloma growth and energy metabolism by repressing cell-cycle and mitochondrial genes following proteasome inhibition. If correct, we would expect HDAC3 to occupy genes repressed by proteasome inhibitors and HDAC3 DNA association to increase following treatment. To test this, we performed HDAC3 ChIP-seq experiments in MM.1S cells. In support of this notion, we found that 58.65% of sites with reduced H3K27 acetylation following proteasome inhibition are associated with HDAC3 DNA occupancy (Fig. 4A). In addition, we found increased HDAC3 DNA association at the *c-MYC* super-enhancer and at the promoters of c-MYC target genes upon treatment with proteasome inhibitors. Importantly, proteasome inhibition did not increase global cellular levels of HDAC3 protein (Fig. 4B–E; Supplementary Fig. S4A and S4B). This finding indicates that DNA-associated HDAC3 levels are locally controlled with spatial specificity by the proteasome and show site-specific increases following proteasome inhibition. As exemplified with the *AKAP1* and *AURKB* gene promoters, reduced H3K27 acetylation colocalized with increased HDAC3 DNA association after proteasome inhibition (Supplementary Fig. S4B). Proteasome inhibition did not modify the abundance of other class I HDACs (HDAC1 and HDAC2) at the promoters of cell-cycle genes (Supplementary Fig. S4C).

To test whether HDAC3 directly deacetylates H3K27 following proteasome inhibition, we generated a stable, inducible MM.1S-shHDAC3 knockdown cell line after screening for HDAC3 knockdown efficiency and confirming specificity (Supplementary Fig. S4D–S4F). ChIP-seq assays showed that acetylation of H3K27 at cell cycle/mitochondrial gene promoters and super-enhancers of genes relevant for multiple myeloma biology (*c-MYC*, *BCL-XL*, *CCND2*, *IRF4*, *MCL1*, *PIM1*, *PRDM1*, and *XBP1*) was mildly increased in HDAC3 knockdown cells compared with nonsilencing cells. Moreover, when treated with proteasome inhibitors, the loss of H3K27 acetylation was significantly attenuated when HDAC3 was knocked down compared with nonsilencing multiple myeloma cells (Fig. 4F; Supplementary Fig. S5). Peak summits were up to 40% higher in HDAC3 knockdown cells after treatment, indicating that the repressive effect of proteasome inhibitors is, at least partially, dependent on HDAC3. To test the effect of lower epigenetic repression on transcription, we measured mRNA expression of cell-cycle and mitochondrial genes in MM.1S-shHDAC3 cells after proteasome inhibition compared with treated nonsilencing cells (Fig. 4G). The results confirm that the gene-suppressive effect of proteasome inhibition is reduced in HDAC3 knockdown cells. Also, reduction of HDAC3 alone was sufficient to promote basal mitochondrial respiration and ATP production in MM.1S cells (Supplementary Fig. S4G). These data indicate that gene repression through proteasome inhibition is mediated by HDAC3. In addition, these studies demonstrate that proteasome inhibition stabilizes HDAC3 locally at promoters and super-enhancers to rapidly repress target genes. Global HDAC3 levels are not affected by proteasome inhibition.

### The SIAH2 Ubiquitin Ligase Antagonizes HDAC3-mediated Repression in Multiple Myeloma Cells

The results indicate that the spatially restricted turnover of HDAC3 at defined chromatin sites dictates activity of genes important for multiple myeloma growth. To examine DNA-associated protein turnover, we mapped and quantified poly-ubiquitination after exposing cells to a brief pulse with a proteasome inhibitor (16). Performing ChIP for ubiquitin in cells before and after treatment allows distinguishing nondegradative from degradative ubiquitination. Indeed, when we measured the degradation of DNA-associated proteins, we found clear enrichment at HDAC3 (57.1%) and c-MYC (79.3%) binding sites (Supplementary Fig. S6A and S6B). Functionally, a significant portion of genes repressed by proteasome inhibition showed combined association with c-MYC and HDAC3 (Supplementary Fig. S6C). The association of c-MYC and HDAC3 DNA occupancy with protein turnover was also evident at the promoters of c-MYC target genes and at the c-MYC super-enhancer (Supplementary Fig. S6D and S6E). Gene transcription is a dynamic process and the local quantities of regulatory proteins are controlled through cyclic binding to and removal from DNA. This cycle is at least partially driven by proteasome-dependent protein elimination (44). Our work demonstrates that HDAC3 is stabilized at the promoters of cell-cycle and mitochondrial genes and at the *c-MYC* super-enhancer, suggesting that HDAC3, or factors recruiting HDAC3 to DNA, are targeted for proteasomal degradation. To address whether c-MYC overexpression, in turn, can overcome the epigenetic block of HDAC3, we generated MM.1S cells transduced with a fully functional fluorescently labeled copy of c-MYC (45). The cells expressed c-MYC at 3.1-fold higher levels, but showed no significant change in viability following bortezomib treatment (Supplementary Fig. S6F). These results indicate that elevated c-MYC levels are not sufficient to overcome the epigenetic block created by proteasome inhibition.

**FIGURE 5:**
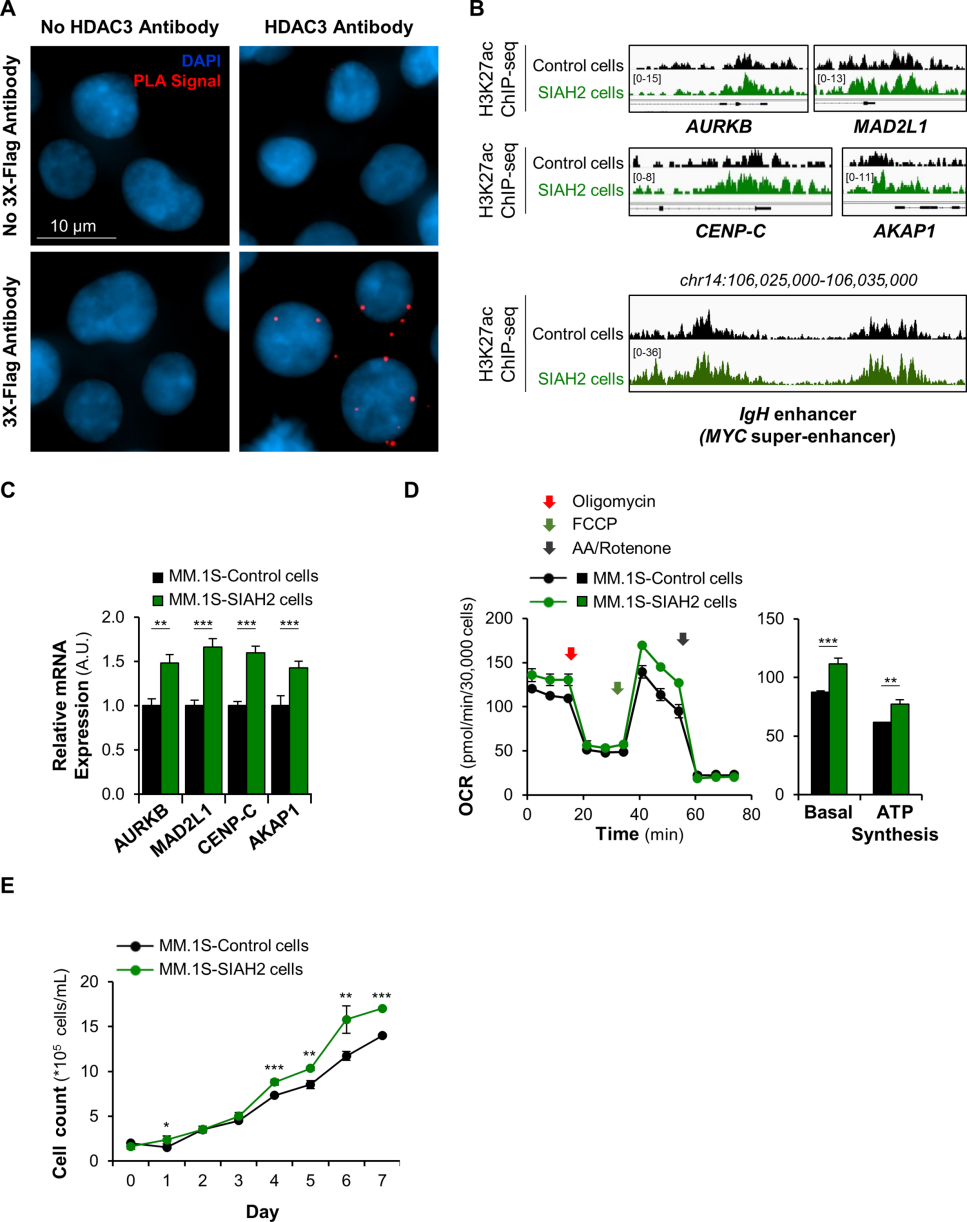
The Seven In Absentia Homolog 2 (SIAH2) ubiquitin ligase antagonizes HDAC3-mediated repression in multiple myeloma cells. **A,** Detection of HDAC3-SIAH2 interactions by proximity ligation assay (PLA) in MM.1S-3xFlag-tagged SIAH2 cells cultured in presence of 0.5 μmol/L lactacystin to prevent auto-degradation of the instable ubiquitin ligase (55). Nuclei are shown stained blue with DAPI and PLA signals represented as red dots. **B,** Representative IGV browser ChIP-seq tracks of H3K27ac peaks at the promoter of cell-cycle and mitochondrial genes and the *c-MYC* super-enhancer show up to 2-fold increased H3K27 acetylation in the MM.1S-SIAH2–overexpressing cell line. The IGV tracks are represented at the same scale (numbers in brackets at the *y*-axis). For the four gene top panels, the gene structure is shown in black at the bottom of each panel and the genomic region on the *x*-axis spans 2.5 kb. IGV snapshots are representative of two independent experiments. **C,** qRT-PCR analysis of HDAC3 target genes shows increased transcription in MM.1S-SIAH2—overexpressing cells. ***, *P* < 0.001; **, *P* < 0.01; and *, *P* < 0.05 determined by unpaired Student two-tailed *t* test. **D,** Measurement of mitochondrial respiration in MM.1S-SIAH2–overexpressing cells. Basal OCR values and ATP synthesis were measured as described in Supplementary Fig. S3D legend. ***, *P* < 0.001; **, *P* < 0.01 determined by unpaired Student two-tailed *t* test. **E,** Cell growth curves show that SIAH2-overexpressing MM.1S cells proliferate faster than control cells over a period of 8 days. ***, *P* < 0.001; **, *P* < 0.01; and *, *P* < 0.05, SIAH2 group compared with control group, determined by unpaired Student two-tailed *t* test. Cell viability was not affected by SIAH2 overexpression.

The ubiquitin ligase SIAH2 has previously been reported to target HDAC3 (46–48) and its recruiting factor NCoR1 for degradation (49). When analyzing *HDAC3* and *SIAH2* transcript expression profiles in 1,457 cancer cell lines, including 27 multiple myeloma cell lines, in the Cancer Cell Line Encyclopedia (50), we found that high levels of *SIAH2* were associated with low *HDAC3* expression. This anti-correlation was specifically observed in multiple myeloma cell lines, but not in other hematopoietic cell lines or in a bulk analysis covering all cell lines (Supplementary Fig. S7A), suggesting an antagonistic regulation of these two factors in multiple myeloma cells. Further supporting a potential involvement of SIAH2 as an antagonist to HDAC3, the ubiquitin ligase has oncogenic potential in several malignancies (51–54). The intracellular levels of SIAH2 protein are generally low, due to rapid SIAH2 auto-ubiquitination and subsequent degradation, making biochemical studies of this ubiquitin ligase challenging (55). To determine whether SIAH2 and HDAC3 interact at the protein level, we performed proximity ligation assays in multiple myeloma cells expressing tagged SIAH2 (Supplementary Fig. S7B), following stabilization by proteasome inhibitors (Supplementary Fig. S7C; ref. 56). An imaging-based analysis showed that SIAH2 interacts with HDAC3 protein (Fig. 5A) and this interaction was confirmed by SIAH2 pull-down assays where we detected HDAC3 protein in the immunoprecipitated fraction (Supplementary Fig. S7D). These combined results indicate that SIAH2 can target HDAC3 for degradation.

Next, we evaluated the impact of SIAH2 on gene regulation, histone acetylation, and cell physiology. qRT-qPCR assays and genome-wide ChIP-seq analysis of H3K27ac profiles showed that overexpression of SIAH2 elevated H3K27 acetylation up to 2-fold and significantly enhanced mRNA transcription of HDAC3-target genes (Fig. 5B and C). Gene ontology analysis of promoters with increased H3K27 acetylation in SIAH2-overexpressing cells showed enrichment for cell division and nuclear-encoded mitochondrial genes (Supplementary Fig. S7E). In addition, we performed functional assays to test cell proliferation and mitochondrial activity in SIAH2-overexpressing cells. These cells showed increased oxidative phosphorylation and ATP production compared with control cells (Fig. 5D). Furthermore, SIAH2-overexpressing cells proliferated faster than control cells (Fig. 5E) without impact on cell viability. These results suggest that SIAH2 facilitates HDAC3 removal from the promoters of cell-cycle and mitochondrial genes, de-represses transcription, and enhances mitochondrial activity and proliferation in multiple myeloma cells.

## Discussion

Proteasome inhibitors, along with immunomodulators and monoclonal antibodies, are the backbone of multiple myeloma treatment. Despite being more than 20 years in use, the molecular mechanisms that make these drugs so effective remain elusive. As pleiotropic drugs, proteasome inhibitors affect many different cellular pathways, including pro- and antiapoptotic pathways (57). Specifically, how proteasome inhibition impacts epigenetics and transcription in multiple myeloma is one of its least understood aspects. We performed an integrative genomic analysis and discovered that proteasome inhibition rapidly blocks transcription of *c-MYC* and approximately 2,000 target genes enriched for cell-cycle mediators and nuclear-encoded mitochondrial genes. We found that transcription of these genes is blocked early in the process through epigenetic silencing and reduced chromatin accessibility. H3K27 acetylation, which initiates decondensation of chromatin, is quickly and effectively reduced in the presence of proteasome inhibitors. This loss of euchromatin is particularly evident in promoters of oncogenic genes and in the super-enhancers driving *c-MYC* and other genes that are relevant for multiple myeloma proliferation. As a result, proteasome inhibitors initially stabilize the c-MYC protein by preventing its degradation, but eventually decrease its levels by blocking transcription of the short-lived proto-oncogene. This paradoxical loss of c-MYC has been observed in multiple myeloma, Hodgkin lymphoma, and in the c-MYC–addicted B-cell neoplasm Burkitt lymphoma, although its mechanistic explanation remained elusive (58–60).

A common theme among several B-cell neoplasms, including multiple myeloma, is their dependence on the proto-oncogene *c-MYC* for progression (33–35, 61). Because multiple myeloma is a c-MYC–driven malignancy, several experimental therapies are being tested to reduce c-MYC levels (62). *MYC* activation is generally driven by dysregulation of upstream signaling pathways, gain/amplification of this oncogene, or chromosomal rearrangements involving the *MYC* locus (34, 62). Specifically, elevated *c-MYC* expression driven by translocation of the gene to the immunoglobulin or related enhancers is observed in 15%–50% of patients with multiple myeloma (63–65). Activation of the c-MYC transactivation domain leads to rapid degradation of c-MYC by the ubiquitin–proteasome pathway, which allows RNA polymerase II to unmoor from the promoter and engage in transcriptional elongation (11, 66–68). Despite stabilizing c-MYC protein, proteasome inhibitors, therefore, also block transcription of its target genes (68). In addition, a recent report suggests that patients with elevated *c-MYC* expression show enhanced sensitivity to proteasome inhibitors in relapsed/refractory multiple myeloma (69), indicating that c-MYC might be a clinically relevant target of proteasome inhibition.

Blunting the oncogene c-MYC is one of the prime goals of experimental therapies in multiple myeloma and other c-MYC–dependent cancers (62, 70, 71). For instance, the bromodomain and extraterminal (BET) inhibitor JQ1 is a promising inhibitor of the *c-MYC* super-enhancer and pharmacokinetically improved versions of this drug are entering clinical trials (37, 72). JQ1 prevents the recognition of acetylated histones by chromatin readers. Our results reveal that proteasome inhibitors might have a similar effect on the *c-MYC* super-enhancer by reducing its acetylation. However, while proteasome inhibition reduces transcription of c-MYC, overexpression of the oncogene is not sufficient to overcome this block. Furthermore, it has been noted that c-MYC repression can create chemo-resistant cell clones by inducing a diapause-like state (73). The overall clinical impact of our mechanistic findings, therefore, remains to be elucidated.

Genes that are under control of the proteasome undergo cyclical activation and repression, which involves the exchange of distinct protein complexes through degradation (74). We established the first comprehensive map of HDAC3 DNA occupancy in dependence of proteasome inhibitors in multiple myeloma cells and identified the histone deacetylase HDAC3 as a candidate protein that is either directly or indirectly (i.e., through a recruiting factor because the enzyme is lacking a DNA binding domain) targeted by the proteasome in a site-specific manner. Patients with high levels of HDAC3 have reduced expression of cell-cycle and mitochondrial genes and increased survival when treated with proteasome inhibitors. Importantly, global HDAC3 levels remain unaltered, consistent with previous reports (60), but DNA-associated HDAC3 increases at defined chromatin sites upon proteasome inhibition. These chromatin sites overlap with proteasome-dependent loss of H3K27 acetylation and high levels of poly-ubiquitination of DNA-associated proteins. While HDAC3 is not the only deacetylase occupying c-MYC target genes (75), genetic depletion of HDAC3 alleviates loss of H3K27 acetylation by proteasome inhibitors, suggesting that HDAC3 is the relevant deacetylase targeted by the proteasome, either directly or through degradation of a recruiting factor because HDAC3 cannot bind DNA by itself. Moreover, we found that SIAH2, a ubiquitin ligase that mediates proteasomal degradation of HDAC3 and its recruiting factor NCOR1 (47, 49), colocalizes with the deacetylase, increases acetylation of c-MYC- and HDAC3-controlled genes, and enhances the oncogenic potential of multiple myeloma cells. While SIAH2 activity is associated with fundamental processes such as cell proliferation and apoptosis in hematologic malignancies (51), its oncogenic role in multiple myeloma was previously unclear. Our global analysis of *SIAH2* transcript expression profiles in about 1,500 cancer cell lines indicates high expression of SIAH2 in multiple myeloma cells, suggesting a possible regulatory role in this cancer. Consistent with our data, elevated SIAH2 expression has been reported in drug-resistant cancer cell lines and malignant tissues compared to healthy tissues (47, 76). Because SIAH2 directly interacts with HDAC3, it may derepress proliferative and metabolic genes and represent a target of clinical interest in multiple myeloma.

We also show that the HDAC3 tightly controls *c-MYC*. HDAC3 and c-MYC interact at the protein level, and the deacetylase has been shown to repress specific c-MYC target genes (77–79). However, our study demonstrates a surprisingly high degree of target gene overlap between these two antagonists, involving 56% of the c-MYC regulon. This indicates that HDAC3 may have a more general tumor-suppressive role in keeping c-MYC target genes at bay. In light of these findings, it appears contradictory that HDAC inhibitors can be beneficial in the treatment of multiple myeloma (80). However, several of the tested HDAC inhibitors are nonselective or impair myeloma cells through increased acetylation of proteins outside the nucleus or through nonautonomous effects on the microenvironment (80–83). In addition, even at the level of chromatin, HDAC inhibition does not globally increase gene expression. Optimized transcription requires balanced chromatin modifications, and hyperacetylation — just as deacetylation — represses gene activity (84). Likewise, HDACs can negatively and positively regulate transcription (85). HDAC inhibitors can affect the c-MYC protein as well. Inhibition of HDACs, including HDAC3, destabilizes c-MYC by triggering ubiquitin-mediated degradation of the acetylated oncogene (78, 79, 86). Taken together, these findings point to a pleiotropic function of HDAC3, in which the deacetylase mediates oncogenic or tumor-suppressive effects depending on the biological context and pharmacological agent used. HDAC3-specific inhibitors have not yet been clinically tested. On the other hand, our pharmacogenomic analysis points to a cooperative effect between proteasome inhibitors and elevated HDAC3 expression. Under these conditions, HDAC3 stabilization at defined DNA sites creates an epigenetic block upstream of transcription factor binding that prevents the opening of chromatin.

**FIGURE 6:**
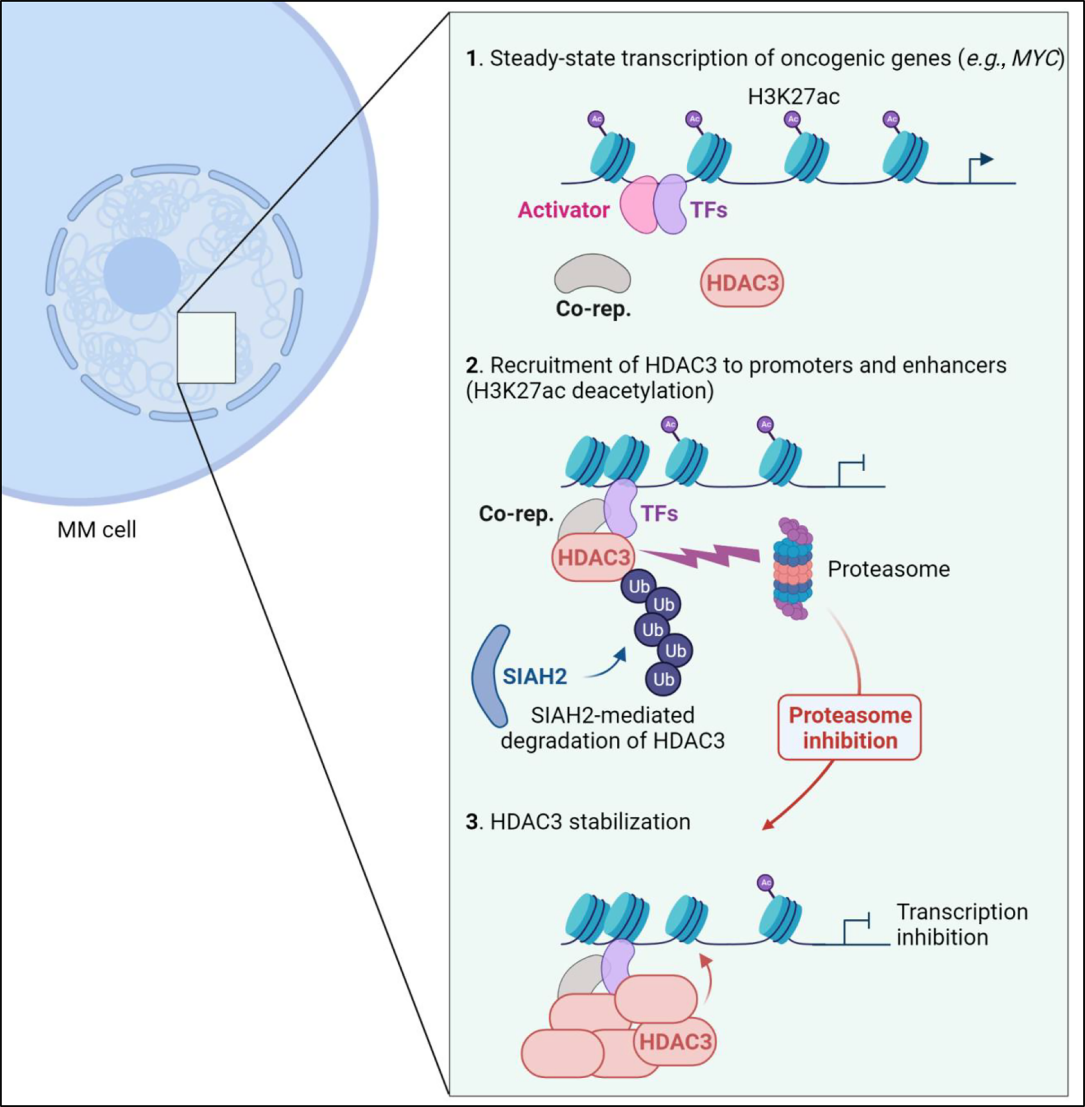
Proteasome inhibitors reshape the chromatin landscape in MM by decreasing H3K27 acetylation at oncogenic promoters and super-enhancers. This epigenetic silencing is mediated by HDAC3, which accumulates at defined genomic sites following proteasome inhibition. The ubiquitin ligase SIAH2 facilitates the removal of HDAC3 from associated promoters and enhancers in the absence of treatment, either through direct degradation (shown) or through removal of a recruiting factor. MM, multiple myeloma; Co-rep, corepressor; TF, transcription factor; Ub, ubiquitin.

To summarize, we define how proteasome inhibition alters the chromatin-associated landscape in multiple myeloma by stabilizing repressor complexes at super-enhancers, including the one controlling *c-MYC*, and at promoters of genes driving proliferation and metabolism (Fig. 6). Proteasome inhibitors paradoxically reduce levels of the short-lived c-MYC protein and impair c-MYC activity by disrupting H3K27 acetylation and increasing chromatin condensation. Our pharmacogenomic analysis suggests that this effect is mediated by HDAC3 and patients with elevated expression of this enzyme might show enhanced benefits when treated with proteasome inhibitors.

In conclusion, our study contributes to a better understanding of the epigenetic and transcriptional vulnerabilities that render multiple myeloma cells sensitive to proteasome inhibition. To our knowledge, this is the first research to comprehensively address how reduced protein degradation directly impacts the turnover of DNA-associated proteins, with consequences for gene activity and multiple myeloma cell growth. Our results indicate that proteasome inhibitors are potent antagonists of the c-MYC regulon, highlight the transcriptional dynamics between gene activators and repressors in multiple myeloma, and open potential avenues for personalized treatment options involving epigenetic modifiers. Interfering with the UPS in a more targeted manner to stabilize DNA-associated HDAC3 and antagonize c-MYC may result in more potent and less toxic therapeutics, leading to improved patient survival.

## Supplementary Material

Supplementary Data SD1 RNA-seq data for Volcano plot Fig. 1A

Figure S1 Genome-wide analysis of acute effects of proteasome inhibition on transcription and chromatin in MM cells.

Figure S2 Proteasome inhibition down-regulates cell cycle and mitochondrial gene expression in three different MM cell lines.

Figure S3 Proteasome inhibition represses cell proliferation and mitochondrial activity in MM.

Figure S4 DNA site-specific stabilization of HDAC3 by proteasome inhibition in MM cells.

Figure S5 HDAC3 mediates repression of the H3K27ac mark at super-enhancers of genes relevant for MM biology following proteasome inhibition.

Figure S6 Association of c-MYC and HDAC3 DNA occupancy with protein turnover at the promoters of c-MYC target genes and at the c-MYC super-enhancer.

Figure S7 The SIAH2 ubiquitin ligase antagonizes HDAC3-mediated repression in MM cells.
