# Supplementary material for: Proteasome Inhibitors Silence Oncogenes in Multiple Myeloma through Localized Histone Deacetylase 3 Stabilization and Chromatin Condensation
Source: Cancer Res Commun. 2022 Dec 27;2(12):1693–710. doi: 10.1158/2767-9764.CRC-22-0255 (PMC9949381; doi:10.1158/2767-9764.CRC-22-0255)
Supplement: Figure S1 — Genome-wide analysis of acute effects of proteasome inhibition on transcription and chromatin in MM cells. [file crc-22-0255-s02.pdf]

Figure S1

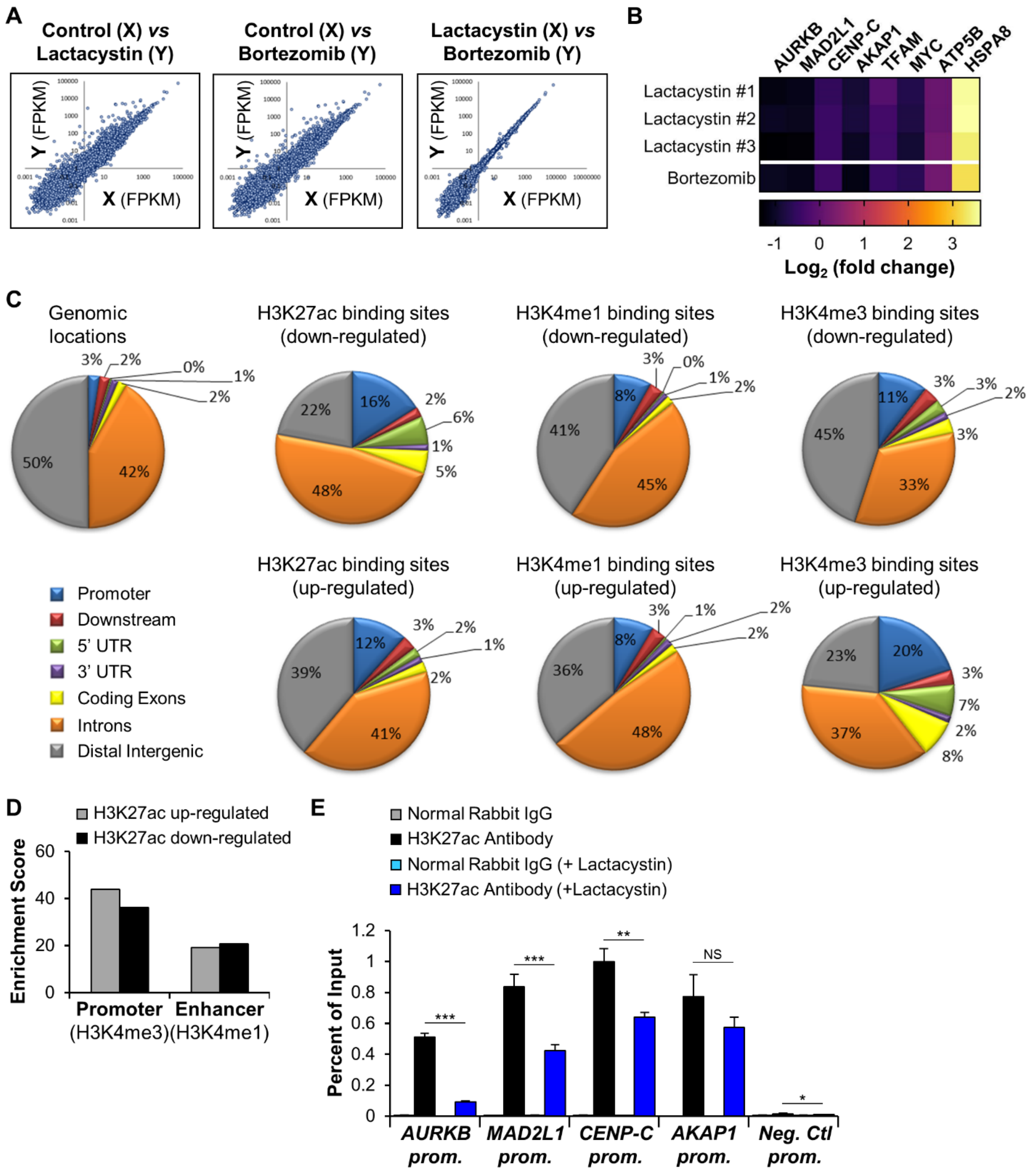

A
Control (X) vs Lactacystin (Y)
Control (X) vs Bortezomib (Y)
Lactacystin (X) vs Bortezomib (Y)
Y (FPKM)
X (FPKM)
B
AURKB
MAD2L1
CENP-C
AKAP1
TFAM
MYC
ATP5B
HSPA8
Lactacystin #1
Lactacystin #2
Lactacystin #3
Bortezomib
-1
0
1
2
3
Log2 (fold change)
C
Genomic locations
H3K27ac binding sites (down-regulated)
H3K4me1 binding sites (down-regulated)
H3K4me3 binding sites (down-regulated)
H3K27ac binding sites (up-regulated)
H3K4me1 binding sites (up-regulated)
H3K4me3 binding sites (up-regulated)
Promoter
Downstream
5' UTR
3' UTR
Coding Exons
Introns
Distal Intergenic
D
H3K27ac up-regulated
H3K27ac down-regulated
Enrichment Score
Promoter (H3K4me3)
Enhancer (H3K4me1)
E
Normal Rabbit IgG
H3K27ac Antibody
Normal Rabbit IgG (+ Lactacystin)
H3K27ac Antibody (+Lactacystin)
Percent of Input
AURKB prom.
MAD2L1 prom.
CENP-C prom.
AKAP1 prom.
Neg. Ctl prom.
NS

Figure S1. Genome-wide analysis of acute effects of proteasome inhibition on transcription and chromatin in MM cells.

A, The transcriptional effects of proteasome inhibition are comparable between the two inhibitors lactacystin and bortezomib. Pairwise scatter plots showing the correlation between the transcriptional targets of lactacystin and bortezomib after RNA-seq expression analysis in MM.1S cells. FPKM counts for all genes in samples treated with lactacystin and bortezomib or in respective control samples are plotted on a log-transformed scale.

B, RNA-seq heatmap showing differentially expressed cell cycle and mitochondrial genes upon treatment of MM.1S cells with proteasome inhibitors lactacystin or bortezomib.

C, Pie charts depicting the genome-wide patterns of histone modifications induced by proteasome inhibition in MM.1S cells. Following treatment with lactacystin, genomic location distribution and percentage of modulated H3K27ac, H3K4me1, and H3K4me3 epigenetic marks for each genomic location category are shown. Data are representative of two independent experiments.

D, Enrichment of H3K27ac mark near promoters (H3K4me3) and enhancers (H3K4me1) following treatment with lactacystin. H3K27 acetylation changes following proteasome inhibition are more common at promoter regions than enhancer regions in MM.1S cells. Data shows the overlap of H3K27ac peaks up- or down-regulated by lactacystin with H3K4me1 or H3K4me3 regions and is represented as the fold increase of altered H3K27ac sites at enhancers/promoters compared to random chromosomal regions. Data from a representative experiment out of two independent experiments are shown.

E, ChIP-qPCR analysis of H3K27ac DNA binding activity at selected promoters following proteasome inhibition with lactacystin in MOLP-8 cells. ***$p<0.001$, **$p<0.01$, and *$p<0.05$ determined by unpaired Student’s two-tailed t-test.
