# Supplementary material for: Proteasome Inhibitors Silence Oncogenes in Multiple Myeloma through Localized Histone Deacetylase 3 Stabilization and Chromatin Condensation
Source: Cancer Res Commun. 2022 Dec 27;2(12):1693–710. doi: 10.1158/2767-9764.CRC-22-0255 (PMC9949381; doi:10.1158/2767-9764.CRC-22-0255)
Supplement: Figure S2 — Proteasome inhibition down-regulates cell cycle and mitochondrial gene expression in three different MM cell lines. [file crc-22-0255-s03.pdf]

Figure S2

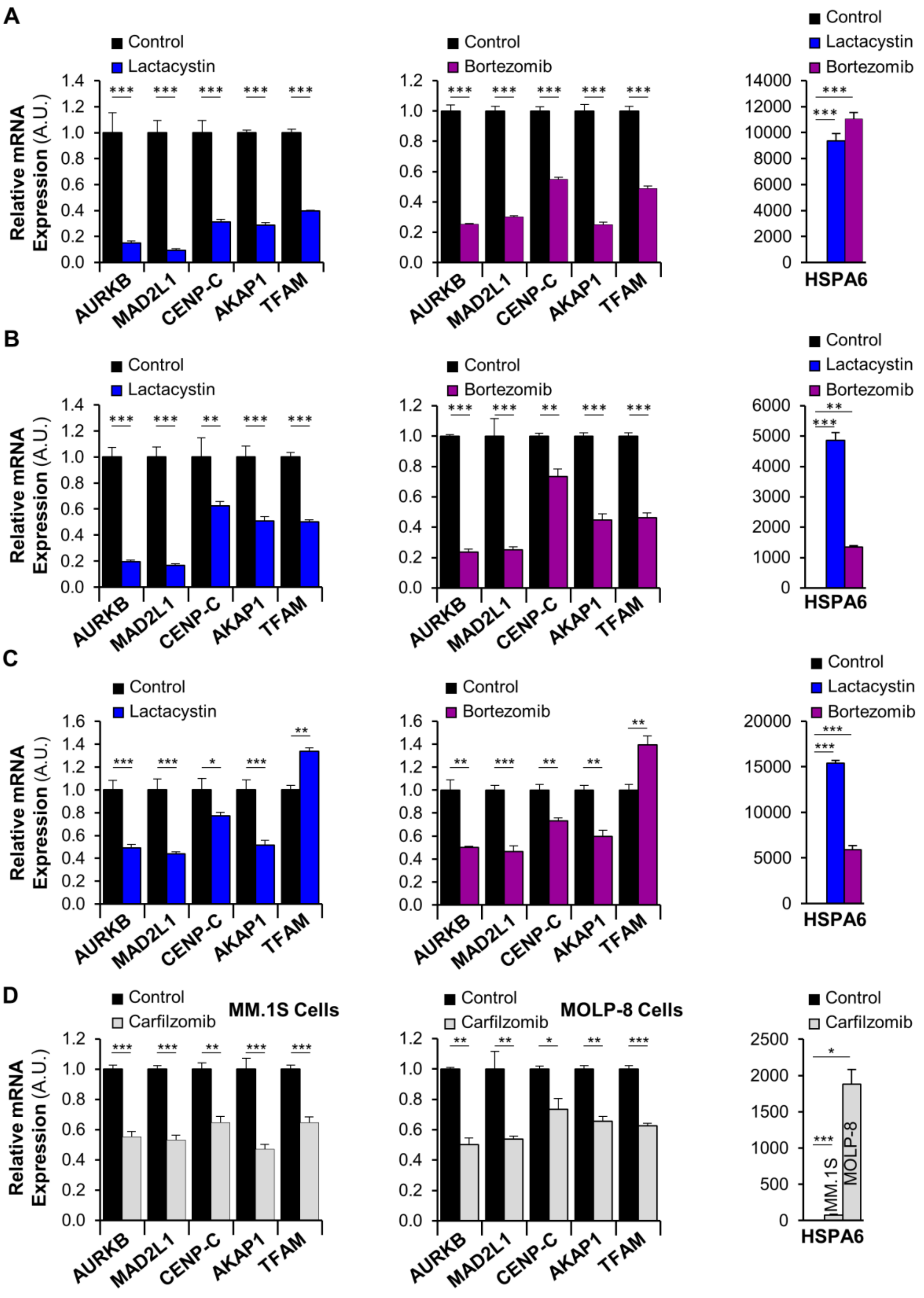
A
B
C
D
Control
Lactacystin
Bortezomib
Carfilzomib
MM.1S Cells
MOLP-8 Cells
Relative mRNA Expression (A.U.)
AURKB
MAD2L1
CENP-C
AKAP1
TFAM
HSPA6
MM.1S
MOLP-8

Figure S2. Proteasome inhibition down-regulates cell cycle and mitochondrial gene expression in three different MM cell lines.

RT-qPCR analysis of genes related to cell cycle, mitochondria and stress response after 6 h treatment of MM.1S cells (**A**), MOLP-8 cells (**B**), and U266.B1 cells (**C**) with 6 µM lactacystin or 60 nM bortezomib. With the sole exception of TFAM, inhibition of the proteasome activity by either lactacystin or bortezomib consistently decreased the mRNA expression levels of the selected genes. ***$p<0.001$, **$p<0.01$, and *$p<0.05$ determined by unpaired Student's two-tailed t-test.

D, RT-qPCR analysis of genes related to cell cycle, mitochondria and stress response after 6 h treatment of MM.1S cells and MOLP-8 cells with 20 nM and 15 nM carfilzomib, respectively. ***$p<0.001$, **$p<0.01$, and *$p<0.05$ determined by unpaired Student's two-tailed t-test.
