# Supplementary material for: Proteasome Inhibitors Silence Oncogenes in Multiple Myeloma through Localized Histone Deacetylase 3 Stabilization and Chromatin Condensation
Source: Cancer Res Commun. 2022 Dec 27;2(12):1693–710. doi: 10.1158/2767-9764.CRC-22-0255 (PMC9949381; doi:10.1158/2767-9764.CRC-22-0255)
Supplement: Figure S3 — Proteasome inhibition represses cell proliferation and mitochondrial activity in MM. [file crc-22-0255-s04.pdf]

Figure S3

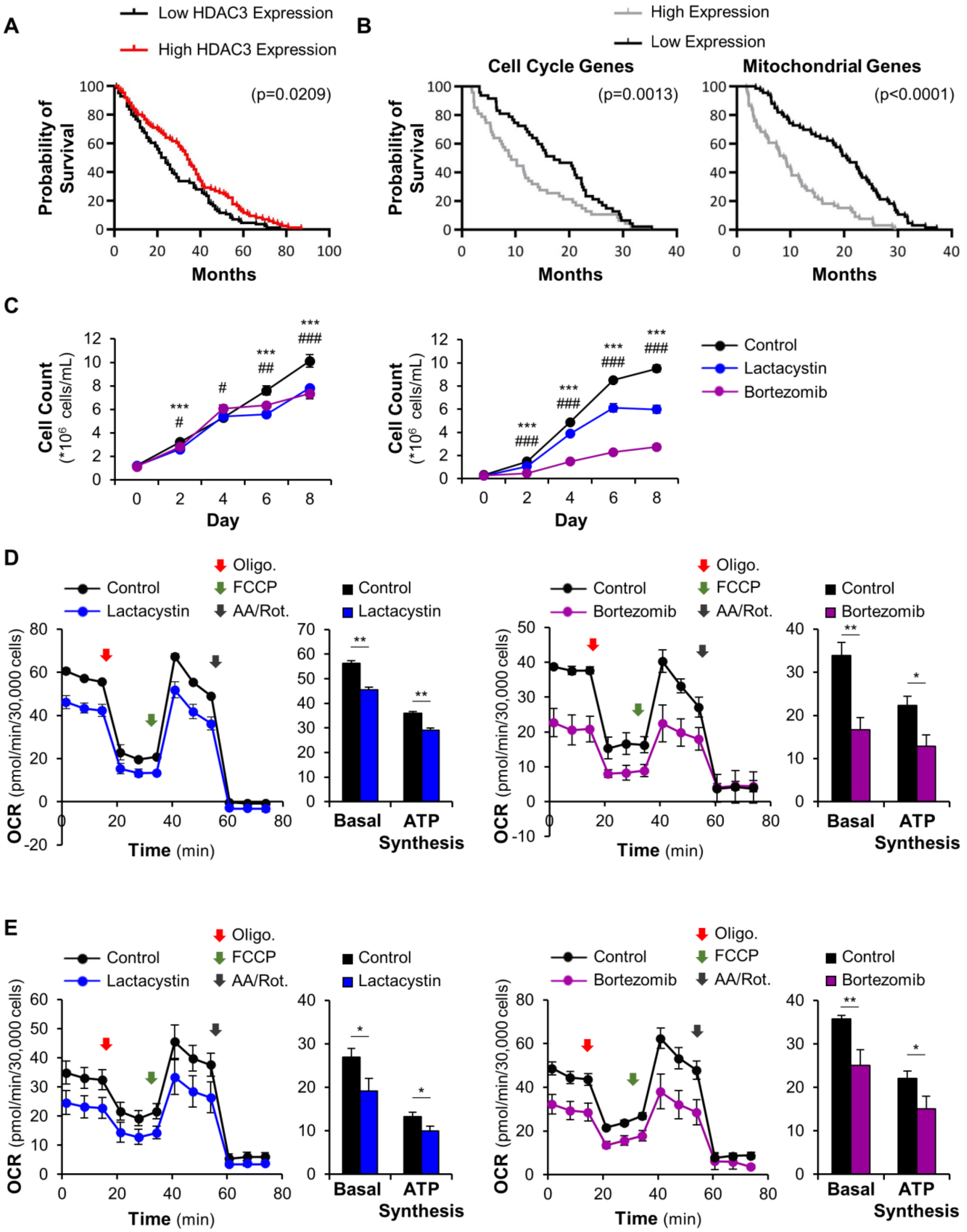
A
Low HDAC3 Expression
High HDAC3 Expression
Probability of Survival
(p=0.0209)
Months
B
High Expression
Low Expression
Cell Cycle Genes
(p=0.0013)
Mitochondrial Genes
(p<0.0001)
C
Cell Count (*10^6 cells/mL)
Day
Control
Lactacystin
Bortezomib
D
Oligo.
FCCP
AA/Rot.
OCR (pmol/min/30,000 cells)
Time (min)
Basal
ATP Synthesis
E

Figure S3. Proteasome inhibition represses cell proliferation and mitochondrial activity in MM.

A, Kaplan-Meier overall survival curves for MM patients according to expression levels of HDAC3 gene. High expression of HDAC3 was associated with better survival in patients treated with aggressive TT2 and TT3 therapies. The median survival time was 23 and 34 months for the low expression and the high expression group, respectively. HDAC3 expression levels were median-dichotomized for separation and overall survival was plotted based on dataset GSE24080 (1). The indicated p-value was calculated with the Gehan-Breslow-Wilcoxon test and significance was confirmed with the log-rank test.

B, Kaplan-Meier overall survival curves for MM patients according to expression levels of cell cycle and mitochondrial genes. The transcript levels of selected genes were determined based on a DNA microarray study in primary $CD138^{+}$ MM cells previously published (2,3) and survival of patients expressing each gene cluster ranked in the top versus bottom quarter was compared in the cohort receiving bortezomib treatment only. For cell cycle genes, the median survival time was 17.1 and 9.2 months for the low expression and the high expression group, respectively. For mitochondrial genes, the median survival time was 20.5 and 9.2 months for the low expression and the high expression group, respectively. The indicated p-values were calculated with the Gehan-Breslow-Wilcoxon test and significance was confirmed with the log-rank test.

C, Growth curves of MM.1S cells (left panel) and MOLP-8 cells (right panel) treated with sub-lethal concentrations of proteasome inhibitors lactacystin or bortezomib over a period of 8 days. Cell growth was significantly slower for cells treated with proteasome inhibitors. ***p<0.001, lactacystin group compared to control group and $^{\#}$p<0.05, $^{\#\#}$p<0.01, $^{\#\#\#}$p<0.001, bortezomib-treated cells vs control cells determined by unpaired Student's two-tailed t-test.

D, Measurement of oxygen consumption rate (OCR) in MM.1S cells exposed to 0.5 µM lactacystin or 3 nM bortezomib for 24 h. Oligomycin (Oligo.), FCCP, and antimycin A/rotenone (AA/Rot.) were sequentially injected by a Seahorse XFp extracellular flux analyzer to assess mitochondrial bioenergetic response over time. Basal mitochondrial respiration was calculated by subtracting non-mitochondrial respiration rate remaining after antimycin A/rotenone addition. ATP production was derived as the difference between basal respiration and respiration rate after oligomycin injection. **p<0.01 and *p<0.05 determined by unpaired Student's two-tailed t-test.

E, Measurement of oxygen consumption rate (OCR) in MOLP-8 cells exposed to 0.5 µM lactacystin or 3 nM bortezomib for 24 h. Mitochondrial basal respiration and ATP synthesis were measured as described in (C). **p<0.01 and *p<0.05 determined by unpaired Student's two-tailed t-test.

Supplementary References:

1. Shi L, Campbell G, Jones WD, Campagne F, Wen Z, Walker SJ*, et al.* The MicroArray Quality Control (MAQC)-II study of common practices for the development and validation of microarray-based predictive models. Nature biotechnology **2010**;28:827-38

2. Mulligan G, Mitsiades C, Bryant B, Zhan F, Chng WJ, Roels S, *et al.* Gene expression profiling and correlation with outcome in clinical trials of the proteasome inhibitor bortezomib. Blood **2007**;109:3177-88
3. Richardson PG, Sonneveld P, Schuster MW, Irwin D, Stadtmauer EA, Facon T, *et al.* Bortezomib or high-dose dexamethasone for relapsed multiple myeloma. The New England journal of medicine **2005**;352:2487-98
