# Supplementary material for: Proteasome Inhibitors Silence Oncogenes in Multiple Myeloma through Localized Histone Deacetylase 3 Stabilization and Chromatin Condensation
Source: Cancer Res Commun. 2022 Dec 27;2(12):1693–710. doi: 10.1158/2767-9764.CRC-22-0255 (PMC9949381; doi:10.1158/2767-9764.CRC-22-0255)
Supplement: Figure S4 — DNA site-specific stabilization of HDAC3 by proteasome inhibition in MM cells. [file crc-22-0255-s05.pdf]

Figure S4

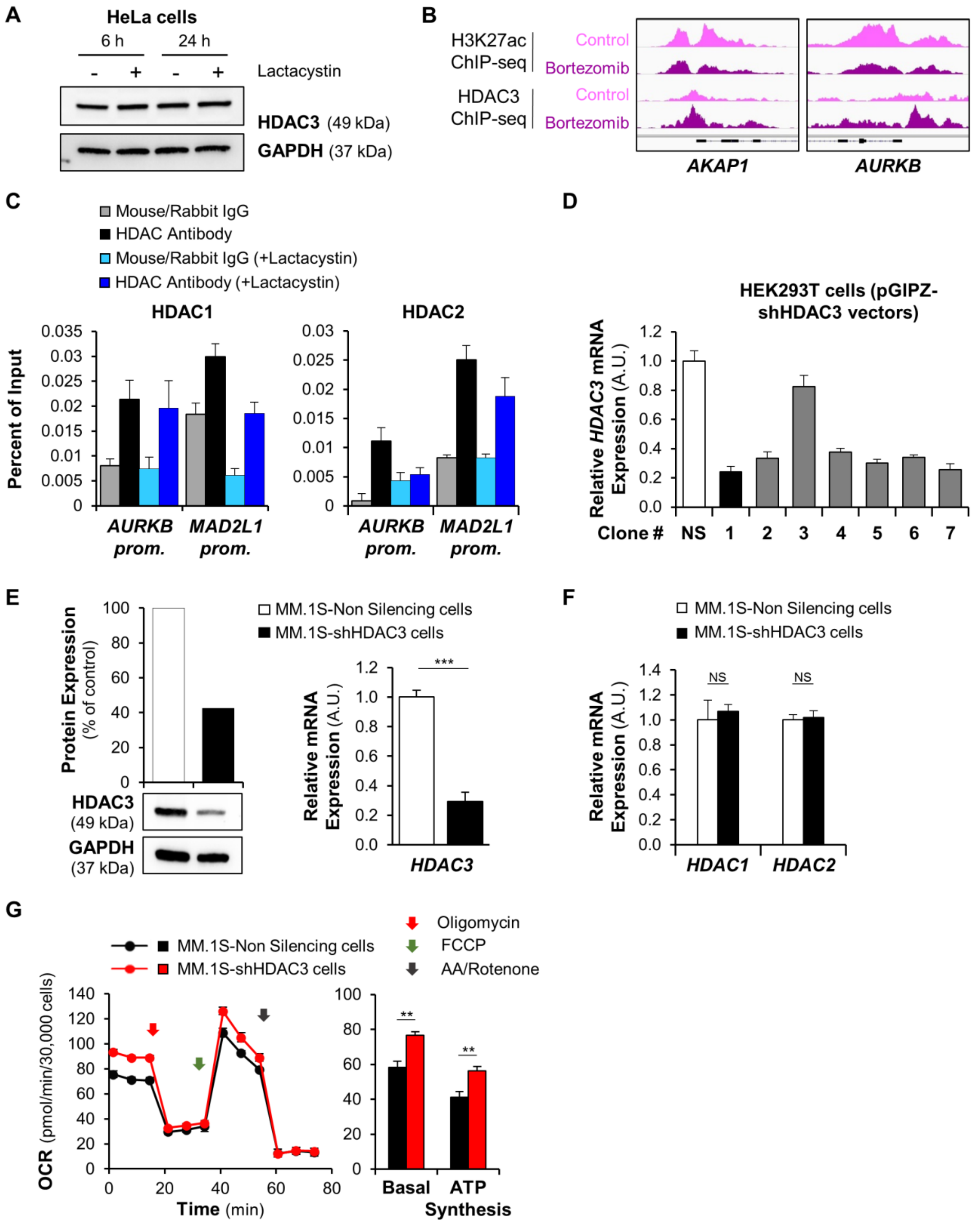

A
HeLa cells
6 h
24 h
- + - +
Lactacystin
HDAC3 (49 kDa)
GAPDH (37 kDa)
B
H3K27ac ChIP-seq
HDAC3 ChIP-seq
Control
Bortezomib
AKAP1
AURKB
C
Mouse/Rabbit IgG
HDAC Antibody
Mouse/Rabbit IgG (+Lactacystin)
HDAC Antibody (+Lactacystin)
HDAC1
HDAC2
Percent of Input
AURKB prom.
MAD2L1 prom.
D
HEK293T cells (pGIPZ-shHDAC3 vectors)
Relative HDAC3 mRNA Expression (A.U.)
Clone # NS 1 2 3 4 5 6 7
E
Protein Expression (% of control)
MM.1S-Non Silencing cells
MM.1S-shHDAC3 cells
HDAC3 (49 kDa)
GAPDH (37 kDa)
Relative mRNA Expression (A.U.)
***
HDAC3
F
MM.1S-Non Silencing cells
MM.1S-shHDAC3 cells
Relative mRNA Expression (A.U.)
NS
NS
HDAC1
HDAC2
G
MM.1S-Non Silencing cells
MM.1S-shHDAC3 cells
Oligomycin
FCCP
AA/Rotenone
OCR (pmol/min/30,000 cells)
Time (min)
**
**
Basal
ATP Synthesis

Figure S4. DNA site-specific stabilization of HDAC3 by proteasome inhibition in MM cells.

A, Western blot analysis of HDAC3 expression levels in HeLa cells after treatment with 6 µM or 0.5 µM lactacystin for 6 h or 24 h, respectively. GAPDH was used as an internal control.

B, ChIP-seq tracks of H3K27ac and HDAC3 occupancy on *AKAP1* and *AURKB* promoters following treatment of MM.1S cells with bortezomib. The gene structure is shown in black at the bottom of each panel. IGV browser images are representative of two independent experiments.

C, Proteasome inhibition does not modify the abundance of HDAC1 and HDAC2 at the promoters of cell cycle genes. ChIP-qPCR analysis of HDAC1 and HDAC2 DNA occupancy at selected promoters following proteasome inhibition with lactacystin in MM.1S cells. The presented data were the result of one experiment.

D, Screening for effective HDAC3 knockdown clones in HEK293T cells. HDAC3 expression levels were measured by RT-qPCR to determine knockdown efficiency. Clone#1 showed the best knockdown efficiency and was selected for further knockdown experiments. Clone #7 was excluded due to cell toxicity. The Open Biosystems nomenclature and clone sequences were the following:

Clone#1: Open Biosystems Clone ID#200204565, Sequence 5'- GCCTGACAATGGTACCTAT-3'
Clone#2: Open Biosystems Clone ID#200222927, Sequence 5'- ATAGCTATCTGGGACATTA-3'
Clone#3: Open Biosystems Clone ID#200222927, Sequence 5'- ATAGCTATCTGGGACATTA-3'
Clone#4: Open Biosystems Clone ID#200222927, Sequence 5'- ATAGCTATCTGGGACATTA-3'
Clone#5: Open Biosystems Clone ID#200243533, Sequence 5'- AACCAGGTAGTGGACTTCT-3'
Clone#6: Open Biosystems Clone ID#200247565, Sequence 5'- CCACTGTTTTATCCAATA-3'
Clone#7: Open Biosystems Clone ID#200251116, Sequence 5'- AGAGCTTCAATATCCCTCT-3'

E, Validation of HDAC3 knockdown in MM.1S cells (Clone#1). HDAC3 expression levels were measured by Western blot (left panel) or RT-qPCR (right panel) to determine knockdown efficiency. The relative expression of HDAC3 protein was quantified in Image J and normalized to that of GAPDH protein. ***$p<0.001$ determined by unpaired Student's two-tailed t-test.

F, RT-qPCR analysis of HDAC1 and HDAC2 transcripts after 6 h treatment of MM.1S cells with 6 µM lactacystin. Inhibition of the proteasome activity did not modulate the mRNA expression levels of HDAC1 and HDAC2 genes. NS, not significant determined by unpaired Student's two-tailed t-test.

G, Measurement of mitochondrial respiration in MM.1S-shHDAC3 knockdown cells. Basal OCR values and ATP synthesis were measured as described in the **Fig. S3D** legend. **$p<0.01$ determined by unpaired Student's two-tailed t-test.
