# Supplementary material for: Proteasome Inhibitors Silence Oncogenes in Multiple Myeloma through Localized Histone Deacetylase 3 Stabilization and Chromatin Condensation
Source: Cancer Res Commun. 2022 Dec 27;2(12):1693–710. doi: 10.1158/2767-9764.CRC-22-0255 (PMC9949381; doi:10.1158/2767-9764.CRC-22-0255)
Supplement: Figure S5 — HDAC3 mediates repression of the H3K27ac mark at super-enhancers of genes relevant for MM biology following proteasome inhibition. [file crc-22-0255-s06.pdf]

Figure S5

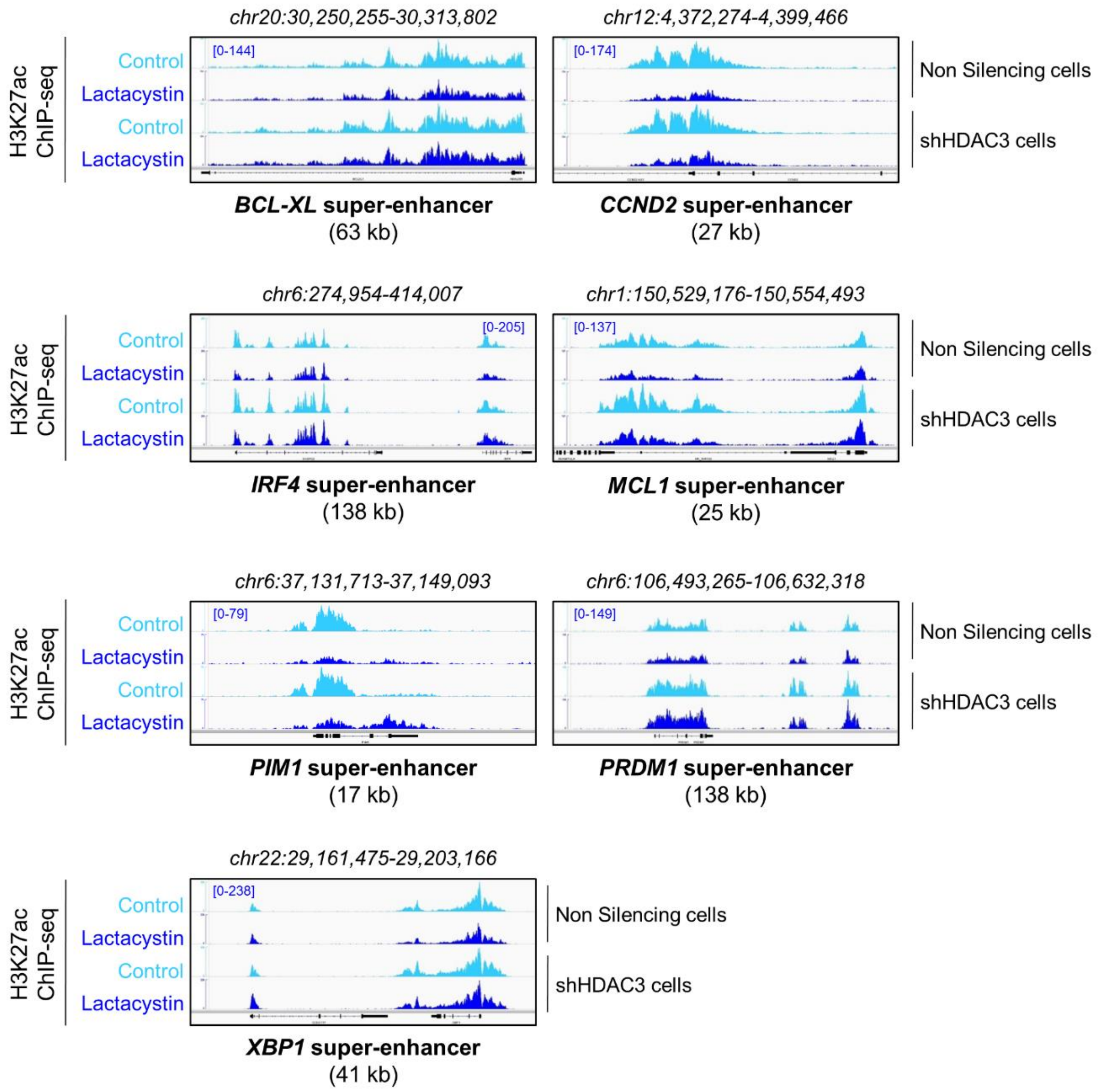

chr20:30,250,255-30,313,802
chr12:4,372,274-4,399,466
H3K27ac ChIP-seq
Control
Lactacystin
Control
Lactacystin
[0-144]
[0-174]
Non Silencing cells
shHDAC3 cells
BCL-XL super-enhancer
(63 kb)
CCND2 super-enhancer
(27 kb)
chr6:274,954-414,007
chr1:150,529,176-150,554,493
[0-205]
[0-137]
IRF4 super-enhancer
(138 kb)
MCL1 super-enhancer
(25 kb)
chr6:37,131,713-37,149,093
chr6:106,493,265-106,632,318
[0-79]
[0-149]
PIM1 super-enhancer
(17 kb)
PRDM1 super-enhancer
(138 kb)
chr22:29,161,475-29,203,166
[0-238]
XBP1 super-enhancer
(41 kb)

Figure S5. HDAC3 mediates repression of the H3K27ac mark at super-enhancers of genes relevant for MM biology following proteasome inhibition.

Gene tracks of H3K27ac ChIP-seq occupancy at super-enhancers in MM.1S-shHDAC3 knockdown cells following exposure to lactacystin. All IGV tracks are represented at the same scale (numbers in brackets at the y-axis). IGV snapshots are representative of two independent experiments. BCL-XL, B-cell lymphoma-extra large; CCND2, Cyclin D2; XBP1, X-box binding protein 1; IRF4, Interferon regulatory factor 4; MCL1, Myeloid cell leukemia sequence 1; PRDM1, PR/SET domain 1.
