# Supplementary material for: Proteasome Inhibitors Silence Oncogenes in Multiple Myeloma through Localized Histone Deacetylase 3 Stabilization and Chromatin Condensation
Source: Cancer Res Commun. 2022 Dec 27;2(12):1693–710. doi: 10.1158/2767-9764.CRC-22-0255 (PMC9949381; doi:10.1158/2767-9764.CRC-22-0255)
Supplement: Figure S6 — Association of c-MYC and HDAC3 DNA occupancy with protein turnover at the promoters of c-MYC target genes and at the c-MYC super-enhancer. [file crc-22-0255-s07.pdf]

Figure S6

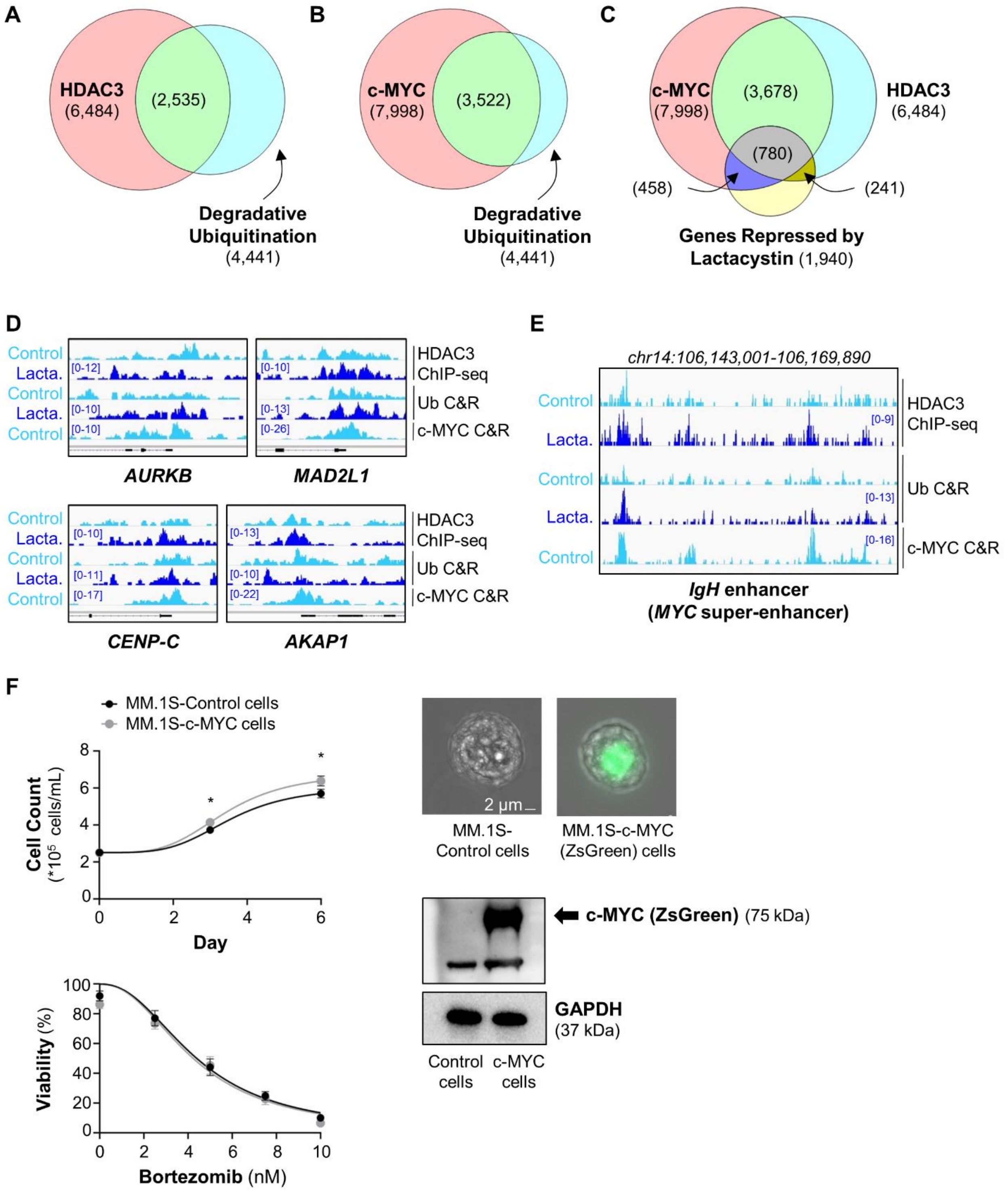
A
HDAC3
(6,484)
(2,535)
Degradative
Ubiquitination
(4,441)
B
c-MYC
(7,998)
(3,522)
Degradative
Ubiquitination
(4,441)
C
c-MYC
(7,998)
(3,678)
HDAC3
(6,484)
(780)
(458)
(241)
Genes Repressed by
Lactacystin (1,940)
D
Control
Lacta.
Control
Lacta.
Control
[0-12]
[0-10]
[0-10]
[0-10]
[0-13]
[0-26]
HDAC3
ChIP-seq
Ub C&R
c-MYC C&R
AURKB
MAD2L1
[0-10]
[0-11]
[0-17]
[0-13]
[0-10]
[0-22]
CENP-C
AKAP1
E
chr14:106,143,001-106,169,890
Control
Lacta.
Control
Lacta.
Control
[0-9]
[0-13]
[0-16]
HDAC3
ChIP-seq
Ub C&R
c-MYC C&R
IgH enhancer
(MYC super-enhancer)
F
MM.1S-Control cells
MM.1S-c-MYC cells
Cell Count
(*10⁵ cells/mL)
8
6
4
2
0
0
2
4
6
Day
*
*
2 µm
MM.1S-
Control cells
MM.1S-c-MYC
(ZsGreen) cells
c-MYC (ZsGreen) (75 kDa)
GAPDH
(37 kDa)
Control
cells
c-MYC
cells
Viability (%)
100
80
60
40
20
0
0
2
4
6
8
10
Bortezomib (nM)

Figure S6. Association of c-MYC and HDAC3 DNA occupancy with protein turnover at the promoters of c-MYC target genes and at the *c-MYC* super-enhancer.

A, Venn diagram showing the significant overlap between HDAC3-associated sites and degradative ubiquitination sites within 1 kb of transcription start site after treatment of MM.1S cells with lactacystin.

B, Venn diagram showing the 79.8% overlap between c-MYC binding sites and degradative ubiquitination peaks within 1 kb of transcription start site after treatment of MM.1S cells with lactacystin.

C, Venn diagram demonstrating significant overlap in c-MYC binding sites, HDAC3-associated sites and gene transcripts repressed by proteasome inhibition within 1 kb of transcription start site in MM.1S cells.

D, Representative IGV tracks of ubiquitin (Ub) binding sites, HDAC3 and c-MYC DNA occupancy show that protein turnover associates with HDAC3 DNA occupancy and c-MYC binding at the promoters of cell cycle and mitochondrial genes following lactacystin exposure of MM.1S cells. All IGV tracks in a given comparison are represented at the same scale (numbers in brackets at the y-axis). The gene structure is shown in black at the bottom of each panel. The genomic region on the x-axis spans 2.5 kb for all the regions. HDAC3 ChIP-seq snapshots are representative of two independent experiments and ubiquitin and c-MYC CUT & RUN tracks were the result of a single experiment. C&R, CUT & RUN; Lacta., Lactacystin.

E, Gene tracks of ubiquitin (Ub) binding sites, HDAC3 and c-MYC DNA occupancy show that protein turnover associates with HDAC3 DNA occupancy and c-MYC binding at the *c-MYC* super-enhancer following lactacystin exposure of MM.1S cells. All IGV tracks in a given comparison are represented at the same scale (numbers in brackets at the y-axis). The genomic region on the x-axis spans 25 kb of the *c-MYC* super-enhancer. HDAC3 ChIP-seq snapshots are representative of two independent experiments and ubiquitin and c-MYC CUT & RUN tracks were the result of one experiment. C&R, CUT & RUN; Lacta., Lactacystin.

F, Cell growth curves show that c-MYC (ZsGreen)-overexpressing MM.1S cells proliferate faster than control cells over a period of 6 days (upper left panel). However, c-MYC overexpression was not sufficient to overcome proteasome inhibition-mediated cell death (bottom left panel). Nuclear c-MYC expression was confirmed by microscopy (N-terminal ZsGreen fluorophore) and total c-MYC expression was an estimated 3.1-fold higher than in control MM.1S cells based on densitometry (right panels). *$p<0.05$, MYC group compared to control group, determined by unpaired Student’s two-tailed t-test.
