# Supplementary material for: Proteasome Inhibitors Silence Oncogenes in Multiple Myeloma through Localized Histone Deacetylase 3 Stabilization and Chromatin Condensation
Source: Cancer Res Commun. 2022 Dec 27;2(12):1693–710. doi: 10.1158/2767-9764.CRC-22-0255 (PMC9949381; doi:10.1158/2767-9764.CRC-22-0255)
Supplement: Figure S7 — The SIAH2 ubiquitin ligase antagonizes HDAC3-mediated repression in MM cells. [file crc-22-0255-s08.pdf]

Figure S7

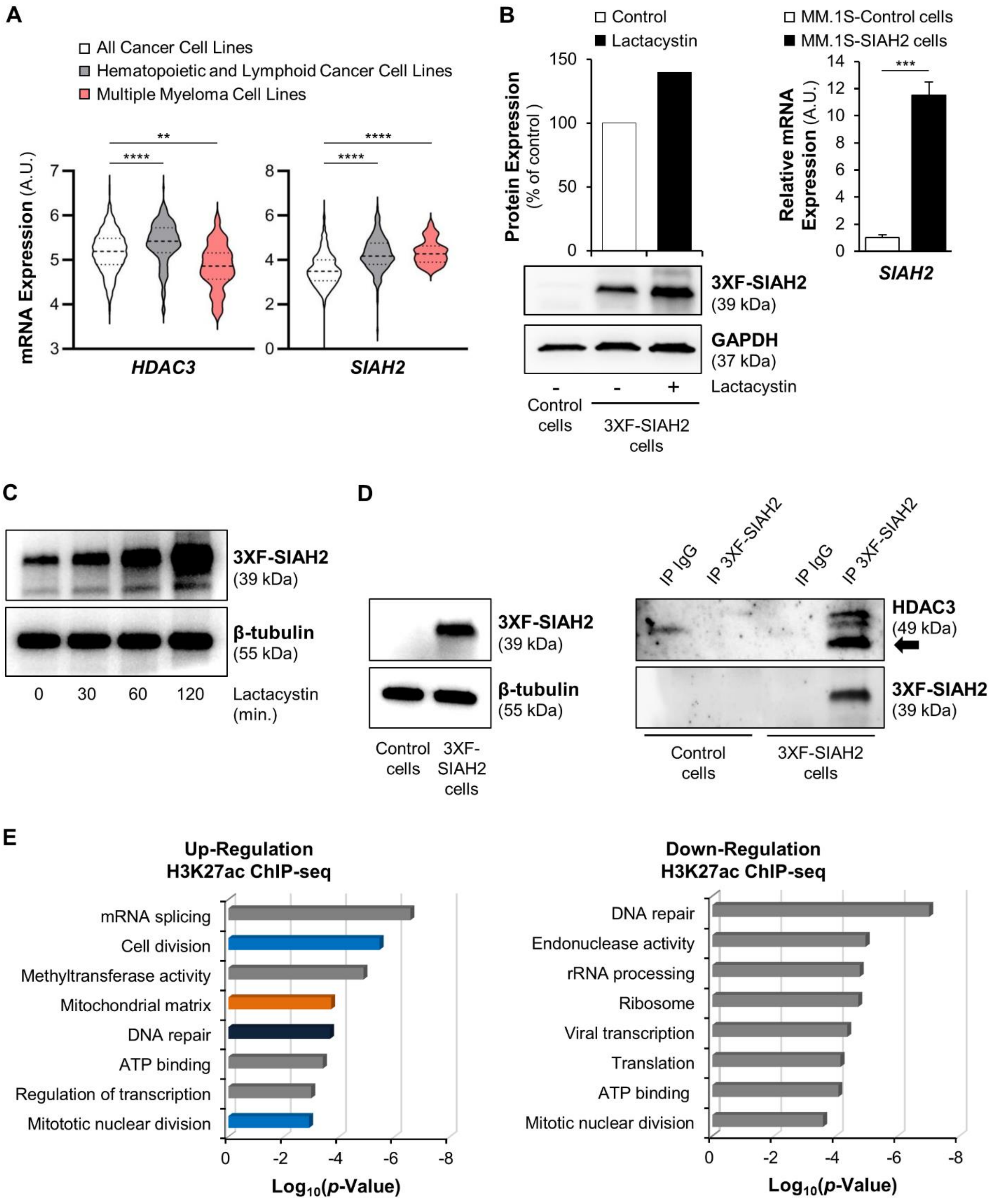

A
All Cancer Cell Lines
Hematopoietic and Lymphoid Cancer Cell Lines
Multiple Myeloma Cell Lines
mRNA Expression (A.U.)
HDAC3
SIAH2
B
Control
Lactacystin
Protein Expression (% of control)
3XF-SIAH2 (39 kDa)
GAPDH (37 kDa)
Lactacystin
Control cells
3XF-SIAH2 cells
MM.1S-Control cells
MM.1S-SIAH2 cells
Relative mRNA Expression (A.U.)
SIAH2
C
3XF-SIAH2 (39 kDa)
β-tubulin (55 kDa)
0 30 60 120
Lactacystin (min.)
D
3XF-SIAH2 (39 kDa)
β-tubulin (55 kDa)
Control cells
3XF-SIAH2 cells
IP IgG
IP 3XF-SIAH2
HDAC3 (49 kDa)
3XF-SIAH2 (39 kDa)
E
Up-Regulation H3K27ac ChIP-seq
mRNA splicing
Cell division
Methyltransferase activity
Mitochondrial matrix
DNA repair
ATP binding
Regulation of transcription
Mitototic nuclear division
Log10(p-Value)
Down-Regulation H3K27ac ChIP-seq
DNA repair
Endonuclease activity
rRNA processing
Ribosome
Viral transcription
Translation
ATP binding
Mitotic nuclear division
Log10(p-Value)

Figure S7. The SIAH2 ubiquitin ligase antagonizes HDAC3-mediated repression in MM cells.

A, *HDAC3* and *SIAH2* transcript levels were compared based on tissue origin in 1457 cancer cell lines. RNA-seq data available in the Cancer Cell Line Encyclopedia (CCLE, Broad Institute) was analyzed and statistical differences were determined by Mann-Whitney test (****$p<0.0001$ and **$p=0.0010$).

B, Validation of SIAH2 overexpression in MM.1S cells. SIAH2 expression levels were measured by Western blot (left panel) or RT-qPCR (right panel) to determine overexpression efficiency. The relative expression of SIAH2 protein was quantified in Image J and normalized to that of GAPDH protein. ***$p<0.001$ determined by unpaired Student's two-tailed t-test.

C, Expression of SIAH2 is increased in MM.1S cells exposed to the proteasome inhibitor lactacystin (25µM). The exposure times are indicated at the bottom of the panel. β-tubulin was used as an internal control. The images were the result of one experiment.

D, SIAH2 and HDAC3 proteins interact in MM.1S cells. Immunoprecipitation of 3XF-SIAH2 in MM.1S cells was followed by Western blot analyses to detect HDAC3 protein and SIAH2 protein (left panel). SIAH2 overexpression was validated by Western blot (left panel). The images were the result a single experiment.

E, Functional distribution of gene clusters enriched in MM.1S-SIAH2 cells. Following ChIP-seq in SIAH2 overexpressing cells, up-regulated or down-regulated H3K27ac epigenetic marks were analyzed for significantly enriched functional annotation terms, as determined by DAVID algorithm. Data are representative of two independent experiments.
